# Acromegaly and cardiovascular disease: mechanisms, clinical impact, and evolving management

**DOI:** 10.1210/endrev/bnag011

**Published:** 2026-04-24

**Authors:** Marta Araujo-Castro, Pedro Marques, Luis Miguel Cardoso, Eider Pascual-Corrales, Julie Refardt, Sebastian J C M M Neggers, José María Jiménez Cassinello, Mónica Marazuela, Manel Puig-Domingo, Betina Biagetti

**Affiliations:** Endocrinology & Nutrition Department, Hospital Universitario Ramón y Cajal, Madrid 28034, Spain; Instituto de Investigación Biomédica Ramón y Cajal (IRYCIS), Madrid 28034, Spain; Pituitary Tumor Unit, Endocrinology Department, Hospital CUF Descobertas, Lisbon 1998-018, Portugal; Faculdade de Medicina, Universidade Católica Portuguesa, Lisbon 1327, Portugal; Department of Endocrinology, Diabetes and Metabolism, Coimbra Local Health Unity, Coimbra 3000-075, Portugal; i3S—Institute for Research and Innovation in Health of the University of Porto, Porto 4200-135, Portugal; IPATIMUP—Institute of Molecular Pathology and Immunology of the University of Porto, Porto 4200-135, Portugal; CIBIT—Coimbra Institute for Biomedical Imaging and Translational Research, Coimbra 3000-548, Portugal; Endocrinology & Nutrition Department, Hospital Universitario Ramón y Cajal, Madrid 28034, Spain; Department of Internal Medicine, Section of Endocrinology, Erasmus Medical Center, Rotterdam 3015, The Netherlands; Department of Internal Medicine, Section of Endocrinology, Erasmus Medical Center, Rotterdam 3015, The Netherlands; Endocrinology & Nutrition Department, Hospital Universitario Ramón y Cajal, Madrid 28034, Spain; Endocrinology & Nutrition Department, Hospital Universitario La Princesa, Madrid 28006, Spain; Endocrinology & Nutrition Department, Hospital Universitario Germans Trias, Barcelona 08916, Spain; Endocrinology & Nutrition Department, Hospital Universitario Vall D’Hebrón and Vall d′Hebron Research Institute (VHIR), Department of Medicine, Autonomous University of Barcelona, Reference Networks (ERN), Barcelona 08035, Spain

**Keywords:** acromegaly, growth hormone, cardiovascular disease, cardiovascular risk factors, management, mortality

## Abstract

Cardiovascular disease is one of the most common and serious complications of acromegaly and a leading contributor to reduced life expectancy in affected individuals. This review examines the prevalence, mechanisms, diagnosis, and management of cardiovascular complications in patients with acromegaly. Growth hormone and insulin-like growth factor 1 excess promotes a range of cardiovascular disturbances, including structural heart changes, arrhythmias, vascular dysfunction, and an increased burden of traditional cardiovascular risk factors such as hypertension, diabetes, and dyslipidemia. Early recognition and targeted treatment of these complications are critical to reducing cardiovascular morbidity and mortality. While surgery and medical therapies for acromegaly aimed at normalizing hormone levels may lead to partial or full reversal of some cardiovascular alterations, many patients require continued management of comorbid conditions to control their overall cardiometabolic risk. Advances in diagnostic strategies and therapeutic options have contributed to improved survival, yet gaps remain in our understanding of how best to prevent or reverse cardiovascular damage in this population. Multidisciplinary care and individualized risk assessment are essential components of modern acromegaly management.

## Essential points

- Cardiovascular complications are the most common and major determinants for the increased morbidity and mortality in acromegaly patients
- An early identification and adequate management of cardiovascular comorbidities is essential to improve patient outcomes and quality of life
- Surgery and medical therapies for acromegaly may lead to partial or full reversal of some cardiovascular alterations, but a residual cardiometabolic risk persisted in many patients
- Multidisciplinary care and individualized risk assessment of the cardiometabolic profile of patients with acromegaly is essential for an optimal management of the disease

Acromegaly is a condition caused by chronic growth hormone (GH) hypersecretion, in most cases due to a benign pituitary adenoma (1). GH stimulates the production of insulin-like growth factor-1 (IGF-1) from the liver and systemic tissues. Both IGF-1 and directly GH have somatic and metabolic effects (1, 2).

The epidemiology of acromegaly is difficult to establish, mostly due to the high heterogeneity of study populations, study designs, and diagnostic/inclusion criteria of acromegaly (3). A recent systematic review and meta-analysis showed an annual incidence of 0.38 cases per 100 000 people (95% CI 0.32-0.44) and a pooled global prevalence of 5.9 per 100 000 people (95% CI 4.4-7.9) (3). In the last decades, an increase in both prevalence and incidence has been reported, which may be explained by increased incidental diagnosis and improved physicians' awareness (4). Acromegaly is usually diagnosed in the fifth decade of life, with an estimated delay of 4.5-5 years (5). At the time of diagnosis, most cases are macroadenomas (>2/3 of cases), which may relate to this diagnostic delay (5). An early detection of the disease is crucial to minimize the likelihood of developing systemic comorbidities associated to GH and IGF-1 excess (6).

In patients with acromegaly, chronically elevated serum GH and IGF-1 levels trigger a systemic syndrome characterized by somatic overgrowth, including physical disfigurement, multiple comorbidities, and premature mortality. Cardiovascular (CV) and metabolic complications are among the most prevalent comorbidities and main mortality drivers in patients with acromegaly (6, 7). Therefore, early identification and adequate management are essential to improve patient outcomes, quality of life, and survival (8). The risk of several CV complications including cardiomyopathy, cardiac valve disease, arrhythmias, and sudden cardiac death seemed to be increased in acromegaly compared to the general population (6). In addition, the prevalence of traditional CV risk factors, such as diabetes, hypertension, and dyslipidemia, is also higher, which further negatively impacts the cardiometabolic profile of these patients (9, 10). Given that GH/IGF-1 excess plays a central role in the development of CV complications in acromegaly, achieving biochemical control is the cornerstone of risk reduction. This can partially reverse some of the structural and functional CV alterations observed in these patients (11-17). Subsequently, optimizing the management of classical CV risk factors such as hypertension, diabetes, dyslipidemia, and obesity remains essential to further reduce the overall CV burden (10).

In this review, we provide an updated overview of acromegaly-related CV and metabolic comorbidities, with a focus on their pathophysiology, epidemiology, and specific clinical characteristics. In addition, we offer practical evidence-based recommendations for the diagnosis and management of such comorbidities.

## Epidemiological and historical context

### Early descriptions of cardiomyopathy in acromegaly

The association between acromegaly and CV complications can be recognized early in medical literature. Pierre Marie's original description of acromegaly in 1886 included cardiac enlargement among a constellation of other features (18). Early 20th-century case series began to document cardiac hypertrophy and heart failure in patients with acromegaly, with autopsy studies revealing significant cardiac enlargement that exceeded what could be attributed to hypertension alone (19). The concept of “acromegalic cardiomyopathy” as a distinct entity emerged later in the 1960s-1970s, when investigators began systematically characterizing the cardiac abnormalities specific to GH excess. Rodrigues et al provided one of the first comprehensive descriptions of the biventricular hypertrophy pattern, distinguishing it from hypertensive heart disease (20). These early observations established that acromegaly could cause intrinsic myocardial disease independent of secondary CV risk factors.

### Evolution of cardiovascular mortality over time in acromegaly

**Figure 1:**
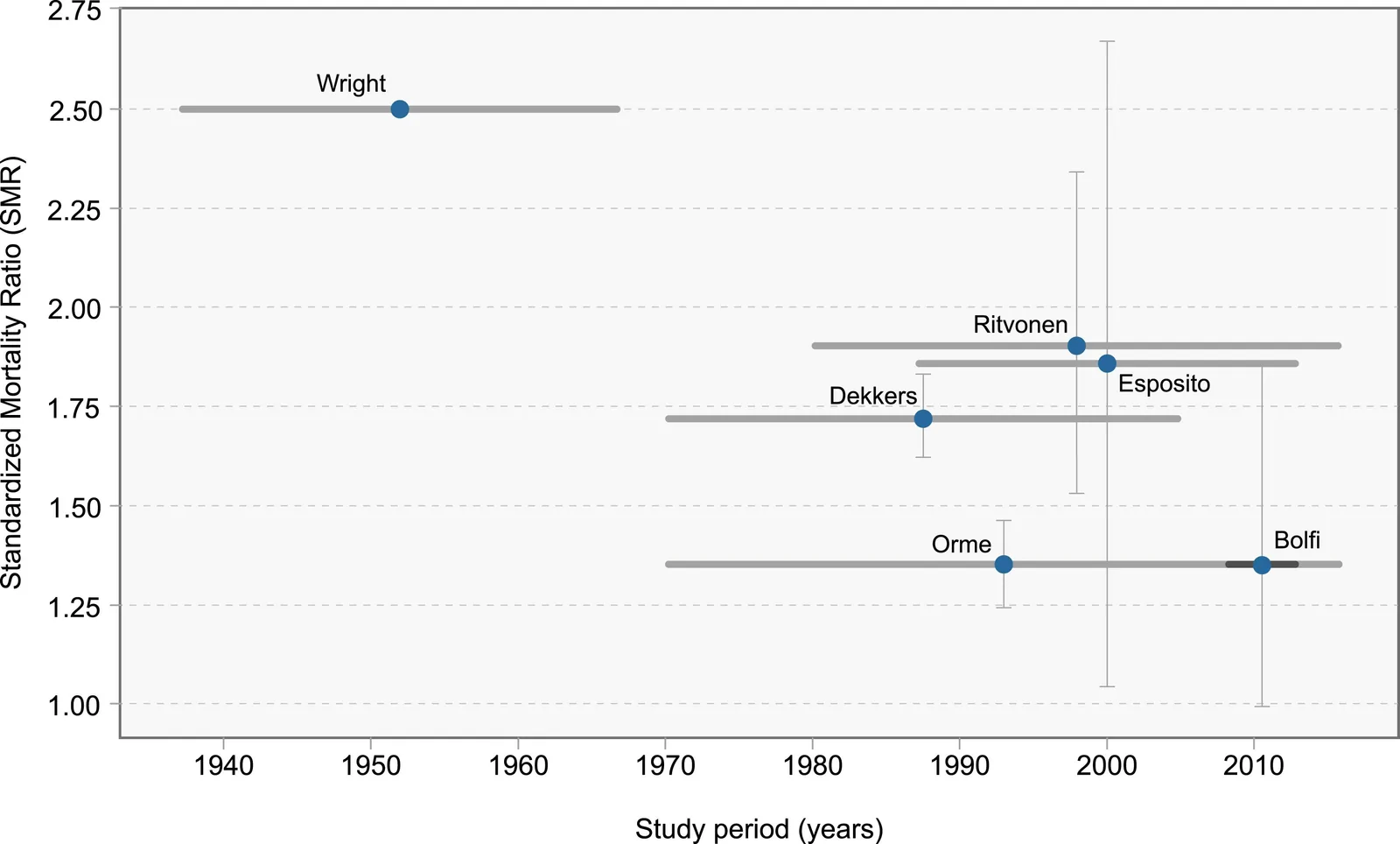
Trend in cardiovascular mortality in patients with acromegaly over time. Standardized mortality ratio (SMR) in acromegaly across historical cohorts according to study period. Each horizontal gray bar represents the time span during which mortality was assessed in each study. Black dots indicate the mid-point of the study period and correspond to the SMR values, with vertical lines representing the 95% CIs, where available. CIs were not reported for the Wright 1970 study. Included studies: Wright (1970) (21), Dekkers (2008; meta-analysis) (22), Ritvonen (2016) (24), Esposito (2018) (25), Bolfi (2018; meta-analysis) (26), Orme (2024) (27). The Dal et al (2016) (23) and L Alexander et al (1980) (28) studies were not included due to the absence of SMR data.

Epidemiological studies from the 1970s-1980s estimated a 2- to 4-fold increase in CV mortality among patients with acromegaly compared to the general population, with CV disease accounting for approximately 60% of excess deaths (21, 22) (Table 1 and Fig. 1). Modern epidemiological understanding of CV risk in acromegaly is increasingly based on large registries and population-based cohorts that provide robust data on contemporary outcomes (26, 29). A Danish national cohort study of 405 acromegaly patients and 4050 age and gender-matched general population controls reported a persistently elevated risk for heart failure (Hazard Ratio [HR] 2.5 (95% CI: 1.4-4.5)) (23). More recent large-scale studies have also shown temporal trends toward improved survival. A comprehensive meta-analysis by Dekkers et al, encompassing 16 studies with over 3000 patients, found an overall standardized mortality risk (SMR) of 1.72 (95% CI: 1.62-1.83), representing a notable improvement from earlier estimates (Table 1 and Fig. 1) (22). More recent data from the Finnish registry (24) suggest continued improvement in CV outcomes, though excess risk persists, with a more noticeable impact on women in line with the data from the Spanish study (30). The Finnish researchers also observed a change in the mortality pattern, which shifted from CV to cancer-related deaths (24). Later in 2018, a systematic review of 42 studies including over 9000 patients found that overall mortality has decreased in acromegaly in parallel with improvements in biochemical control, although CV mortality remained elevated with a pooled SMR of 1.42 (95% CI: 1.28-1.58) (26). Similar trends have been observed in other studies (27, 31), suggesting that therapeutic advances (25, 26, 32) and shorter diagnostic delay (33) have a positive impact on overall acromegaly mortality. More recently, the Danish AcroDEN cohort confirmed a continued decline in CV mortality, with life expectancy in acromegaly now approaching that of the general population (HR: 1.3 [95% CI: 1.0-1.7]) (Table 1 and Fig.1) (34).

**Table 1:** Evolution of cardiovascular mortality in acromegaly

Study	Period	Country	*N*	SMR and 95% CI	Key observations
**Wright et al, 1970** (21)	**1937-1967**	UK	194	2-4 CV deaths ≈60% of total	Seminal evidence of high CV mortality; basis for clinical concern
**Dekkers et al,2008** (22)	**1970-2005**	Meta-analysis	4947	1.72 (1.62-1.83)	First quantitative confirmation of mortality trend improvement
**Dal et al, 2016** (23)	**1991-2010**	Denmark	405	SMR Not reported (HR: 1.3 (95% CI: 1.0-1.7)	Mortality risk remains elevated but uninfluenced by mode of treatment.
**Ritvonen et al, 2016** (24)	**1980-2016**	Finland	333	1.9 (1.53-2.34)	Shift from cardiovascular to cancer-related deaths; greater impact in women.
**Esposito et al, 2018** (25)	**1987-2013**	Sweden	1089	Declined from SMR from 3.45 (2.87-4.02) to 1.86 (1.04-2.67)	Decrease in SMR after 1990; improvement linked to better disease control.
**Bolfi et al, 2018** (26)	**1937-2013** **Before and after 2008**	Meta-analysis	Before 5152 After 5618	Before 1.76 (1.52-2.04 After 1.35 (0.99-1.85)	Reduced total mortality with biochemical control; cancer deaths rising
**Orme et al, 2024** (27)	**1970-2016**	UK	1845	1.35 (1.24-1.46)	Cardiovascular and respiratory diseases were the main causes of death (43% and 19%, respectively).
**Wright et al, 1970** (21)	**1937-1967**	UK	194	2-4 CV deaths ≈60% of total	Seminal evidence of high CV mortality; basis for clinical concern
**Dekkers et al,2008** (22)	**1970-2005**	Meta-analysis	4947	1.72 (1.62-1.83)	First quantitative confirmation of mortality trend improvement
**Dal et al, 2016** (23)	**1991-2010**	Denmark	405	SMR Not reported (HR: 1.3 (95% CI: 1.0-1.7)	Mortality risk remains elevated but uninfluenced by mode of treatment.
**Ritvonen et al, 2016** (24)	**1980-2016**	Finland	333	1.9 (1.53-2.34)	Shift from cardiovascular to cancer-related deaths; greater impact in women.
**Esposito et al, 2018** (25)	**1987-2013**	Sweden	1089	Declined from SMR from 3.45 (2.87-4.02) to 1.86 (1.04-2.67)	Decrease in SMR after 1990; improvement linked to better disease control.
**Bolfi et al, 2018** (26)	**1937-2013** **Before and after 2008**	Meta-analysis	Before 5152 After 5618	Before 1.76 (1.52-2.04 After 1.35 (0.99-1.85)	Reduced total mortality with biochemical control; cancer deaths rising
**Orme et al, 2024** (27)	**1970-2016**	UK	1845	1.35 (1.24-1.46)	Cardiovascular and respiratory diseases were the main causes of death (43% and 19%, respectively).

Evolution of cardiovascular mortality in acromegaly: overview of selected population-based studies, large multicenter cohorts, and meta-analyses reporting standardized mortality rates over time. Studies were selected based on relevance, methodological robustness, geographical and temporal representativeness.

Abbreviations: CI: confidence interval; CV: cardiovascular; SMR: standardized mortality rate.

The age at acromegaly diagnosis has increased in recent decades, partly due to the overall aging of the population and the broader access to imaging techniques. This has resulted in a growing proportion of newly diagnosed patients being older adults (35-37). As age is a well-established risk factor for CV disease, this shift may impact on the profile and prognosis of acromegaly-related complications. Moreover, delayed diagnosis in elderly individuals may be exacerbated by the nonspecific nature of acromegaly symptoms in this population, which may overlap with normal aging, further increasing cardiometabolic burden (38). The increase in life expectancy has been associated with more deaths due to cancer (26), leading to the fact that cancer has become a top cause of death in acromegaly in the last decade.

## Pathophysiology of cardiovascular comorbidities in acromegaly

Chronic GH and IGF-1 excess in acromegaly promotes a wide spectrum of CV phenotypes, including cardiac hypertrophy, diastolic and systolic dysfunction, hypertension, arrhythmias, and valvular disease. Although these complications are often viewed as a unified consequence of somatotropic hypersecretion, experimental and clinical data suggest that GH and IGF-1 act through distinct yet overlapping pathways affecting myocardial growth, fibrosis, and vascular tone. Their specific contributions to each CV phenotype remain difficult to separate in vivo. To facilitate interpretation across phenotypes, we propose an integrative framework organized into four partially overlapping domains: anatomical/structural, hemodynamic, molecular/cellular, and systemic abnormalities. These domains interact dynamically over time, and the relative contribution of each may vary according to disease duration, biochemical control, comorbidities, and age. More detailed information on the pathophysiology of cardiovascular comorbidities in acromegaly is available in Supplementary material.

### Hypertension

**Figure 2:**
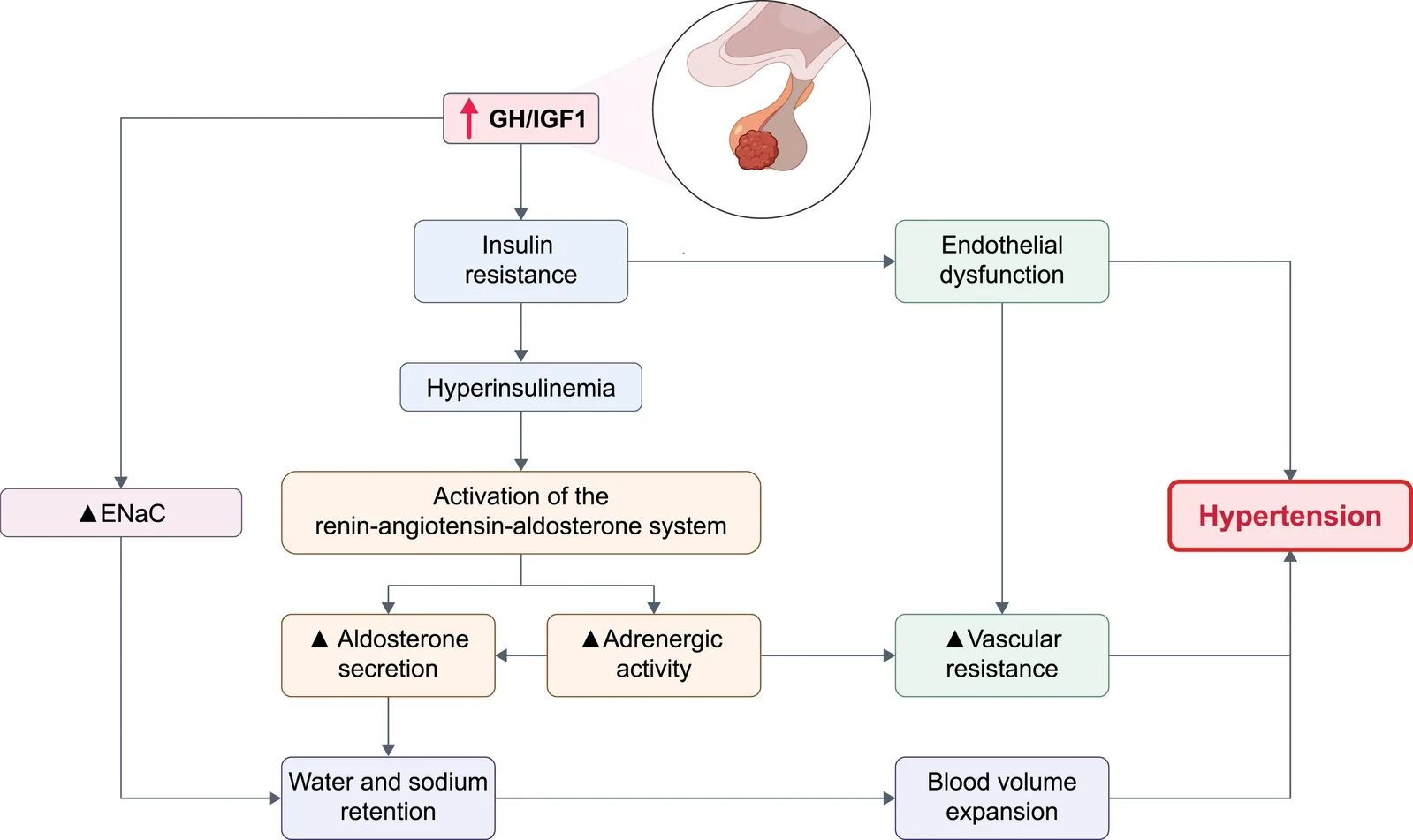
Pathophysiology of hypertension in acromegaly.

The proposed mechanisms in acromegaly-related hypertension include direct effects of GH and IGF-1 in the heart, plasma volume expansion, vascular and insulin resistance, which also impairs endothelial function (39, 40) (Fig. 2). GH and IGF-1 receptors, as well as IGF-1, are expressed in the heart and vasculature, regulating cardiac growth, myocardial contractility, and vascular tone (8, 41). The expansion of plasma volume is a key mechanism leading to hypertension in acromegaly, which results from the sodium and water retention in the distal convoluted tubule induced by excessive levels of GH and IGF-1 (39, 40). Notably, GH stimulates the epithelial sodium channel (ENaC), further enhancing sodium reabsorption and extracellular fluid expansion (42). Another key factor is the stimulation of the renin–angiotensin–aldosterone system by GH (43, 44), which causes adrenergic activation, aldosterone secretion, and increased peripheral vascular resistance (43, 45). It has been proposed that direct activation of the adrenergic system by GH and IGF-1 also contribute to the development of hypertension in acromegaly (Fig. 2) (10, 41, 42, 46-48).

### Acromegaly-related cardiomyopathy

Acromegaly-related cardiomyopathy is defined by the presence of morphological and functional cardiac changes. It typically comprises concentric biventricular hypertrophy, mainly involving the left ventricle, with concomitant diastolic dysfunction occurring in half of cases (8, 49).

The primary mechanism involves the trophic effects of GH and IGF-1 on cardiac myocytes, which have direct anabolic effects on the myocardium, given the expression of GH and IGF-1 receptors in cardiac myocytes (50). In acromegaly-related cardiomyopathy, ventricular walls become concentrically thickened due to an increase in myocyte size, but there are rarely enlarged heart chambers. Additionally, there is increased interstitial fibrosis, which appears to be the main histological feature impairing heart structure and function, increase in interstitial fibroblasts, myofibrillar derangement, and also lympho-mononuclear cell infiltration resembling multifocal myocarditis, which in turn leads to myocyte apoptosis and necrosis (8, 49, 51). The increase in left ventricular mass (LVM) occurs because of a homogeneous increase in both intracellular myocardial mass as well as extracellular myocardial matrix (52).

Although it is known that lipid deposition in cardiomyocytes and GH-specific effects on lipid metabolism and beta-oxidation may aggravate cardiac hypertrophy (50, 53), some authors suggest that there is no evidence of ectopic lipid accumulation in the pathophysiology of the acromegalic cardiomyopathy as they found that after the control of GH excess, myocardial and hepatic lipid content remained unchanged, while pericardial fat was increased in the patients in whom GH excess was controlled (54, 55) In accordance with these findings, it is reported that patients with GH deficiency have an increase of ectopic lipids such as intramyocellular lipids (56).

The evolution of cardiomyopathy in acromegaly follows three stages: (1) the first stage is reversible and is characterized by a biventricular concentric hypertrophy, with increased myocardial contractility and systolic output, and increased heart rate, determining a hyperkinetic syndrome (increased cardiac index); (2) the second stage is characterized by a more pronounced ventricular hypertrophy, associated with a decreased diastolic filling and prolongation of the pre-ejection period at rest, and a systolic dysfunction on effort; and (3) end-stage irreversible cardiomyopathy typically occurs in patients with late diagnosis, uncontrolled or untreated acromegaly, and comprises resting diastolic and systolic dysfunction, low cardiac output, and heart failure (10, 57) (Fig. S1) (58).

Acromegaly-related cardiomyopathy can appear independently of hypertension (10, 49), as supported by reports of about 20% of normotensive young patients with acromegaly developing cardiac hypertrophy (59), and by the demonstration of structural cardiac changes after short-term exposure to GH (60). In this regard, cardiomyopathy can be found in acromegaly patients who do not have hypertension or other CV risk factors (10, 49, 61, 62) Nevertheless, hypertension remains a key factor exacerbating left ventricular hypertrophy (LVH) due to mechanical stress of pressure overload (52, 63) The coexistence of additional CV risk factors, such as dyslipidemia or diabetes, may further accelerate the onset and progression of cardiac complications and outcomes (64).

### Heart valve disease and arrythmias

**Figure 3:**
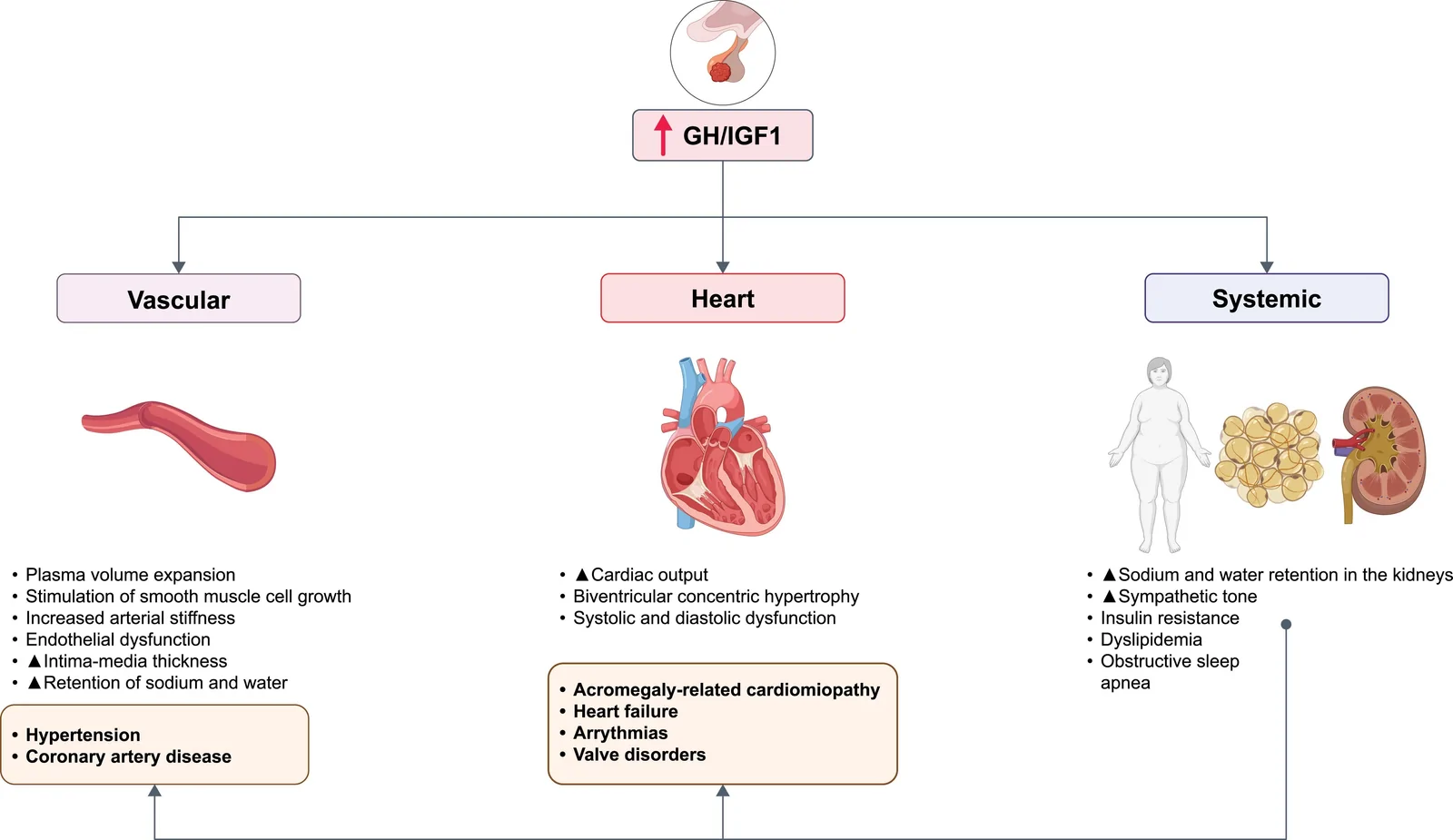
Cardiovascular effects of acromegaly.

Prolonged exposure to high GH and IGF-1 levels leads to increased synthesis of extracellular matrix components and deposition of mucopolysaccharides and collagen in valvular leaflets, resulting in diffuse interstitial fibrosis and myxomatous degeneration of the cardiac valves. This process causes leaflet thickening, redundancy, and impaired coaptation, predisposing to valvular dysfunction, most commonly regurgitation, particularly of the mitral and aortic valves (65-71). Aortic ectasia is also more frequent in patients with acromegaly than in controls (72, 73) (Fig. 3).

Cardiac dysrhythmia in acromegaly may occur because of interstitial fibrosis, myofibrillar derangement and cardiac hypertrophy promoted by IGF-1 and GH excess. These structural alterations, together with pressure overload particularly in hypertensive patients, lead to abnormalities in heart conduction and arrhythmias (70, 74). The pathogenesis is usually multifactorial, including the effects of IGF-1 (75), cardiac structural changes (76), and electrophysiological alterations (increased QT interval variability) (Fig. 3).

### Vascular disease

**Figure 4:**
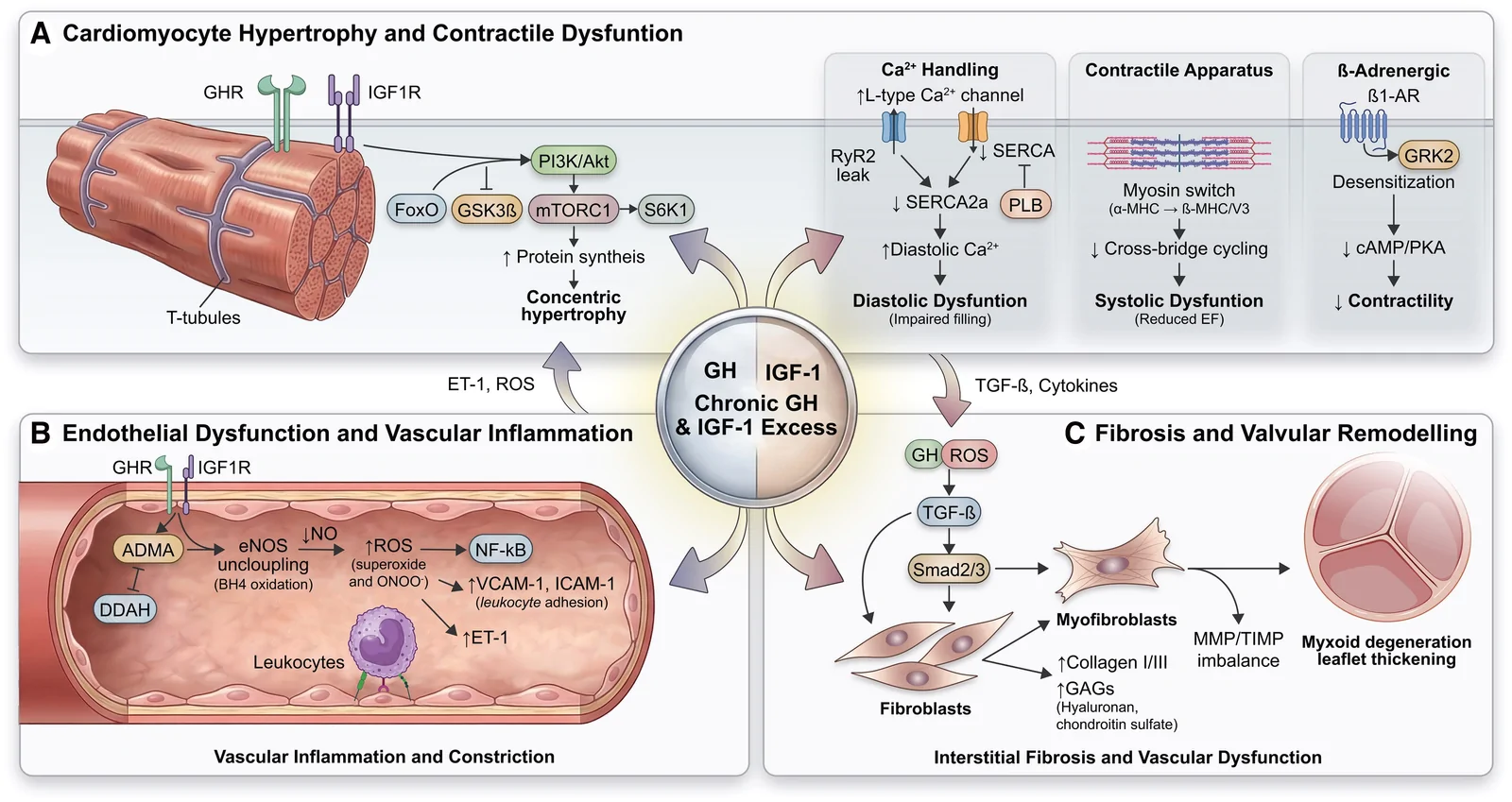
Molecular/cellular mechanisms of cardiovascular complications in acromegaly.

Chronic GH and IGF-1 excess promotes vascular injury through direct and indirect mechanisms, including endothelial dysfunction, smooth muscle proliferation, oxidative stress, inflammation, and extracellular matrix remodeling (42, 77, 78). These alterations lead to early vascular stiffening, accelerated atherosclerosis, and a prothrombotic state, contributing to both coronary artery disease (CAD) and cerebrovascular disease in acromegaly (Fig.3 and Fig. 4).

In acromegaly, GH and IGF-1 excess lead to endothelial dysfunction and atherosclerosis through various mechanisms: (1) increased oxidative stress; (2) increase in pro-inflammatory cytokines; (3) impairment of endothelial repair processes; (4) modification of hemodynamic forces; (5) alteration of vascular morphological through the proliferation of vascular smooth muscle cells; (6) inducing expression of adhesion molecules; and (7) coexistence of CV comorbidities such as hypertension, insulin resistance, diabetes, SAS, dyslipidemia, and metabolic syndrome (79, 80). Despite these GH/IGF-1-related vascular effects and the increased burden of cardiometabolic risk factors, the prevalence of overt CAD and myocardial infarction appears to be lower than anticipated (23, 81-83) potentially due to the vasodilatory and anti-atherogenic properties of physiological IGF-1 (79), and suggesting that the excess of GH *per se* may not be associated with an additional risk for CAD (79, 83). In experimental models, IGF-1 infusion reduced plaque size and oxidative stress, and enhanced endothelial NO bioavailability and anti-inflammatory pathways (84). However, chronic supraphysiological exposure may override these protective effects, leading to subclinical atherosclerosis and vascular dysfunction (79, 85).

Patients with acromegaly show early cerebrovascular involvement, including impaired flow-mediated dilation, increased carotid-femoral pulse wave velocity, and reduced microvascular reactivity, even in the absence of clinical disease (84, 86). Endothelial biomarkers such as von Willebrand factor, VCAM-1, and intercellular adhesion molecule 1 are elevated, reflecting vascular inflammation and damage (87, 88). Additionally, a prothrombotic milieu is frequent, with increased fibrinogen, plasminogen activator inhibitor-1 (PAI-1), and platelet hyperreactivity (Fig. 4 and Table S1) (58, 89). The risk of cerebrovascular events increases with age, disease duration, poor biochemical control, and prior radiotherapy exposure, which is associated with delayed vascular injury, carotid stenosis, and white matter hyperintensities on MRI (8, 90).

### Thrombosis and hypercoagulability

Studies consistently show elevated levels of fibrinogen in acromegaly (77, 89, 91-97). Campello et al (92). described an increase in factor VIII, and significantly enhanced thrombin generation, with this last parameter surprisingly showing a significant inverse correlation with GH and IGF-1 levels, being higher in treated patients, which overall suggests a complex relationship between the disease activity and coagulation dynamics. Alterations of natural anticoagulant agents, such as decreased protein C (89, 95) and its cofactor protein S (89, 94, 95), decreased tissue factor pathway inhibitor (89), as well as increased antithrombin III (89, 95) and fibrinogen have also been described (77, 91, 92). Fibrinolysis may be impaired, due to increased levels of tissue plasminogen activator (89), PAI-1 (89), and thrombin-activatable fibrinolysis inhibitor antigen (22), further contributing to a hypercoagulability and hypofibrinolytic state. However, some studies did not replicate these findings (89, 91, 92, 94, 96), and hemostasis data remains inconsistent (92, 95) and insufficient to draw firm conclusions (Table 2).

**Table 2:** Studies focusing on coagulation and fibrinolysis alterations in acromegaly

Study (year)	Design	*N*	Comparison	Fibrinogen (mg/dL)	Factor VIII (%)	AT III	Protein C (%)	Protein S (%)	PAI-1 (ng/mL)
**Landin-Wilhelmsen et al (1997)** (77)	Comparative	20 ACR 20 controls	20 ACR with active disease vs 20 healthy controls	**↑** 400 (310—610) vs 240 (130 -300) **	n/a	n/a	n/a	n/a	n/a
**Sartorio et al** **(2000)** (96)	Case-control	10 ACR 64 controls	All acromegalic patients vs healthy controls	**↑** 398 ± 111 vs 291 ± 71 **	n/a	n/a	n/a	n/a	**NS**
**Vilar et al (2007)** (94)	Case-control	62 ACR 36 controls	Active disease vs healthy controls	**↑** 434.8 ± 117.6 vs 314.3 ± 65.8 **	n/a	**NS**	**NS**	**↓** 71.9 ± 24.7 vs 99.6 ± 12.9 *	n/a
**Erem et al (2008)** (89)	Case-control	22 ACR 22 controls	Active disease vs healthy controls	**↑** 370.3 ± 81.2 Vs 249.3 ± 14.4 **	**NS**	**↑** 31.9 ± 7.0 vs 24.2 ± 2.2 mg/dL *	**NS**	**↓** 103.1 ± 20.3 vs 119.3 ± 26.7 *	**↑** 72.3 ± 39.7 vs 30.3 ± 8.3 **
**Colak et al (2016)** (95)	Case-control	39 ACR 35 controls	Active disease vs healthy controls	**↑** 436.9 ± 143.7 vs 362 ± 44.9 **	n/a	**↓** 94.5 ± 27.7 vs 113.9 ± 8.3 % **	**↓** 97.3 ± 18.2 vs 123.4 ± 23.1 **	**↓** 81.5 ± 13.2 vs 100.5 ± 14.7 **	n/a
**Amado et al** **(2018)** (97)	Case-control	23 ACR 11 controls	Active disease vs NF pituitary adenomas	**↑** 427.1 ± 61.93 vs 356.91 ± 86.03 *	**NS**	**↑** 1.13 ± 0.09 vs 0.95 ± 0.16 U/mL **	**NS**	**NS**	**NS**
**Campello et al** **(2020)** (92)	Case-control	40 ACR 40 controls	All acromegalic patients vs healthy controls	**↑** 342 (308.8-386.3) vs 314 (256-349) **	**↑** 138 (124 -170) vs 132 (104 -158) *	**NS**	**NS**	**NS**	**NS**
**Kyriakakis et al** **(2020)** (91)	Case-control	40 ACR 40 controls	Active disease vs healthy controls	**↑** 38 ± 2 vs 26 ± 2 **	n/a	n/a	n/a	n/a	**NS**

Values are expressed as means ± standard deviation or as median (interquartile range). Some studies like Vilar et al (94), Amado et al (97), Campello et al (92), and Kyriakakis et al (91) run multiple comparisons between active disease, controlled disease, and controls, in which case only the comparisons between active disease and controls are shown in this table to allow results comparability.

Abbreviations: ↑/↓, significantly increased or decreased; ACR, patients with acromegaly; AT III, antithrombin III; NF, non-functioning; PAI-1, plasminogen activator inhibitor 1; n/a, not assessed; NS, non-significant difference; *P < .05; **P < .01.

### Diabetes Mellitus and dyslipidemia

Excessive GH secretion in patients with acromegaly promotes insulin resistance in the liver and muscle, which can eventually lead to the development of diabetes when the β-cell exhausts its capacity to enhance insulin secretion. The pathogenesis of glucose abnormalities in acromegaly is complex (98-100). In acromegaly-induced diabetes mellitus, insulin resistance seems to be driven mostly by direct and indirect effects of GH. GH is the primary factor, promoting lipolysis and raising free fatty acid levels, while IGF-1 has a minor role in its pathophysiology and conversely, has some insulin-sensitizing effects (101).

An older age, longer disease duration, active disease (high GH/IGF-1 levels), family history of diabetes, increased body mass index (BMI), hypertension and dyslipidemia are well-recognized risk factors for diabetes mellitus in patients with acromegaly. Notably, many cases of acromegaly-associated diabetes mellitus are reversible or may significantly ameliorate after achieving acromegaly biochemical control, but persistent metabolic risk remains elevated (102, 103). Body composition changes, such as increased lean mass, decreased subcutaneous adipose tissue, and increased intermuscular adiposity, as well as the coexistence of several CV risk factors, may further contribute to the adverse pro-diabetogenic metabolic milieu in acromegaly (104).

The lipid disorders in acromegaly mainly include hypertriglyceridemia (approximately 3 times higher than in the general population) and low high-density lipoprotein (HDL) cholesterol levels (11, 105, 106). Acromegaly is also associated with derangements in the lipoprotein metabolism, mainly consisting of increase in circulating lipoprotein(a), Apo A-I, and Apo E (involved in the transport of cholesterol and triglycerides), as well as small dense low-density lipoprotein (LDL) particles (107). The development of dyslipidemia in acromegaly is multifactorial, driven by the direct effects of GH and IGF-1 excess and indirect effects mediated by the underlying metabolic disturbances, especially insulin resistance (78, 108, 109).

### Sleep apnea syndrome

Respiratory complications in acromegaly originate due to structural and functional changes in the entire respiratory system, resulting in sleep apnea syndrome (SAS) and/or respiratory insufficiency (8). Excessive levels of GH and IGF-1 in acromegaly lead to marked anatomical changes mainly concerning the craniofacial bones and soft tissues, including the swelling of the tongue, alterations in the respiratory mucosa and cartilages, lung volume, as well as changes in muscle structure, reduction of lung elasticity and increase in lung distensibility (70). For obstructive SAS, modifications of the facial skeleton, pharynx, and tongue have a key role for its occurrence in patients with acromegaly (110, 111). Small and upper airway narrowing has been described in 35% and 26% of acromegaly patients, respectively (112-114), and overall, SAS may be found in 20-80% of patients with acromegaly (115, 116).

In central SAS, that represents one-third of the cases of SAS in acromegaly, excess GH and IGF-1 impair brain control of respiration (117). Higher IGF-1 levels, male gender, older age, increased BMI, and longer disease duration have been associated with a higher risk of SAS (118). However, other studies identified smoking, female gender, and presence of lung disease as risk factors of more severe SAS, while no correlation was found between SAS and disease duration, serum GH and IGF-1, SRLs treatment, BMI, and associated comorbidities (119). In addition, acromegaly treatment complications, such as hypopituitarism, could be related to obstructive SAS prevalence (120).

## Cardiovascular comorbidities in acromegaly and its distinctive features

### Hypertension

#### Epidemiology

Early studies reported very discrepant prevalences of hypertension in patients with acromegaly, ranging from 18% to 60% (25, 45, 121-130). This wide range may be explained by the different criteria to diagnose hypertension, as well as by the heterogeneous populations across the studies, often not accounting for possible confounding factors, such as age, sex, or ethnicity (25, 121, 123-130). Also, data from studies relying only on a single BP determination to establish the diagnosis of hypertension seem to vastly overestimate the prevalence of hypertension in comparison with 24-hour ambulatory blood pressure monitoring (ABPM) (131, 132).

A recent systematic review showed that CV comorbidities such as myocardial/LVH, diastolic dysfunction, and hypertension are among the most frequent comorbidities, with a reported mean prevalence of 38% (7). A compilation of data from 19 national acromegaly registries encompassing more than 16 000 patients reported hypertension as the most prevalent comorbidity, with a weighted mean prevalence of 35.7%, varying across the registries from 11% to 54% (29). Considering only data from large national registries, summarized in Table 3, the reported prevalence of hypertension in acromegaly ranges from 27% up to 48%. In the Mexican acromegaly registry, the prevalence of hypertension was 27%, but this cohort with more than 2000 patients displayed a relatively young age at diagnosis (mean of 41.1 years) (128). In contrast, the Austrian and French registries reported higher prevalence rates of 47.8% and 47.5%, respectively (129, 130). The Italian (123), the Canadian (124), and the German (125) registries reported intermediate prevalences of 33%, 37%, and 45%, respectively. Recent data from the Iberian (Spain and Portugal) acromegaly registry encompassing 434 acromegaly patients found that 209 (48.2%) had hypertension at the time of acromegaly diagnosis (122) (Table 3). Overall, the data from these large registries suggest that nearly one-third to one-half of acromegaly patients may have hypertension at the diagnosis of acromegaly or during the disease course (25, 121, 123-130).

**Table 3:** Hypertension prevalence in large (>500 cases) national acromegaly registries

Registry: country and year	Study population Size (n)	Prevalence of hypertension (%)	Age at diagnosis (years)	Sex Ratio (F/M)	Mean or median IGF-1 levels
**Italy (2012)** (123)	1512	33.0	45.0	1.42	744 ± 318 ng/mL
**Canada (2013)** (124)	649	37.0	45.0	0.97	311.8 (148.3) % ULN
**Germany (2013)** (125)	1344	45.0	44.5	1.36	NA
**South Korea (2013)** (126)	1350	NA	44.1	1.15	985.4 ± 504.7 µg/L
**UK (2013)** (127)	2572	NA	47.4	0.99	NA
**Mexico (2016)** (128)	2057	27.0	41.1	1.47	678 ng/mL (IQR 533.4-887)
**Austria (2016)** (129)	607	47.8	40.0-45.0	1.18	NA
**France (2017)** (130)	980	47.5	46.0	1.13	NA
**Sweden (2018)** (25)	1089	NA	51.6	1.14	NA
**Spain and Portugal (2025)** (122)	434	48.2	50.4	1.40	3.0 ± 3.11 above the ULN

This table summarizes the prevalence of hypertension and clinical features in national acromegaly registries and large multicenter studies including over 500 patients. The data shown depicts the variability of hypertension prevalence and demographic features across the different registries. Hypertension diagnosis was based on office blood pressure measurements. “NA” indicates data not available in the source registry.

Abbreviations: F/M, female/male; IGF-1, insulin-like growth factor 1; IQR, interquartile range; ULN, upper limit of normality.

#### Screening and diagnosis

Acromegaly patients with hypertension typically show a pattern characterized by higher diastolic and lower systolic BP and a higher prevalence of non-dippers when compared to patients without acromegaly (133-135). Hypertension tends to occur at early ages and appears to be unrelated to gender or family history (133). In addition, the morning BP surge was reported to be higher (ie, 29 mmHg) when compared with patients without acromegaly (ie, 19 mmHg), and this correlated with higher IGF-1 levels (136).

Hypertension is one of the most relevant negative prognostic factors for CV mortality (137-140). In the ACROSTUDY, the presence of hypertension was associated with a higher BMI, diabetes, hyperlipidemia, CV disease, and increased mortality, when compared with acromegaly patients without hypertension (13). Therefore, early detection and appropriate management of hypertension are crucial in acromegaly to reduce CV morbidity and mortality (141).

**Figure 5:**
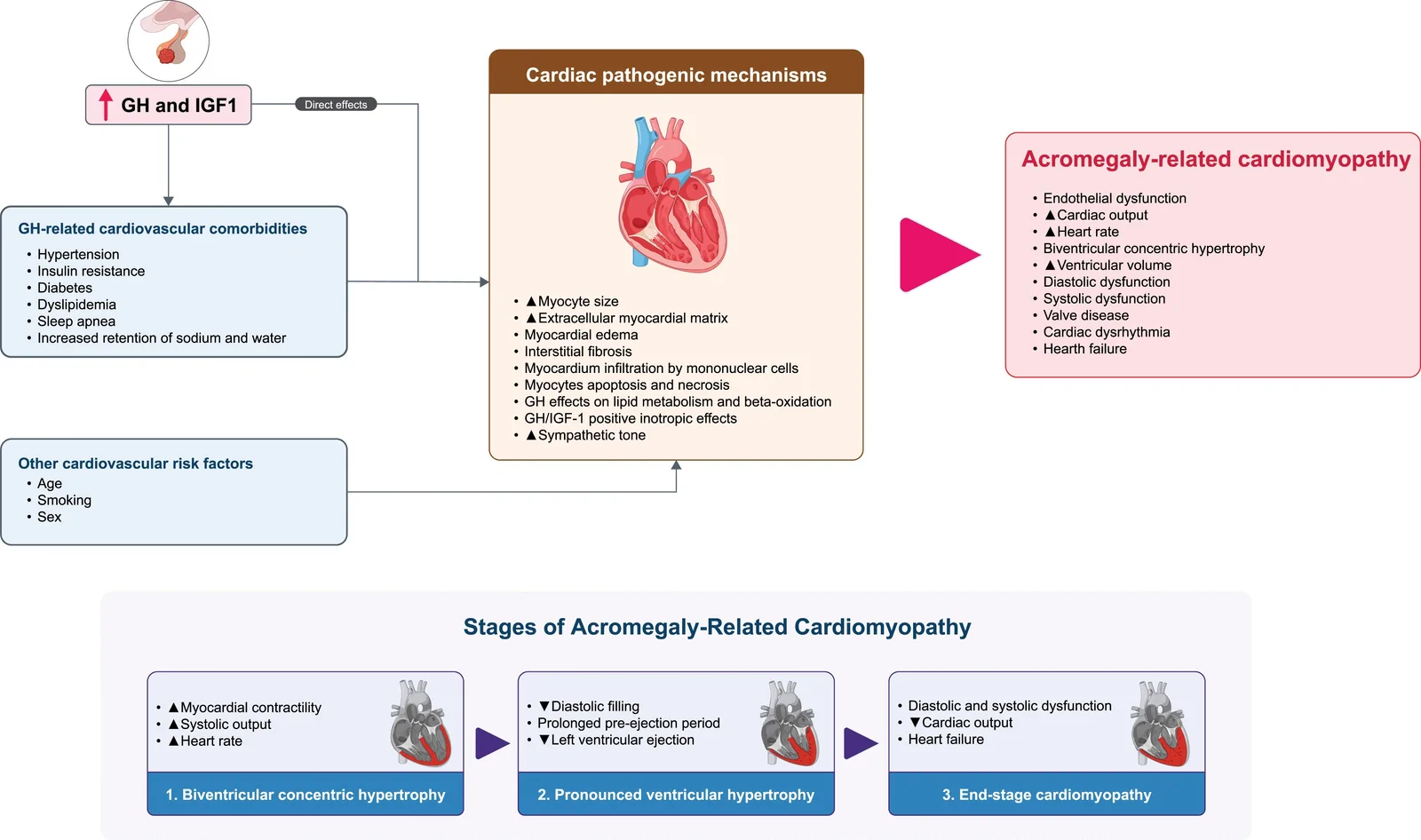
Suggested approach for screening and diagnosing hypertension in acromegaly. ABPM, ambulatory blood pressure monitoring; ACR, albumin to creatinine ratio; BP, blood pressure; ECG, electrocardiography; eGFR, estimated glomerular filtration rate; HBPM, home blood pressure monitoring; LVH, left ventricular hypertrophy. These recommendations are in accordance with the guidelines form the general population (142).

Given the often insidious onset of hypertension in acromegaly and its high frequency, active screening for hypertension is recommended at diagnosis and then periodically (eg, every 6 months); for patients under anti-hypertensive treatment, monitoring is also advised every 6 months or after titration of antihypertensive drugs (Table S2 and Fig. 5) (58, 141). A more comprehensive assessment of hypertension-related end-organ damage may also be necessary in acromegaly, particularly if specific CV complications are present or suspected (Table S2) (58).

In the absence of specific studies, the current diagnosis criteria of hypertension are the same as for the general population, which means an office BP ≥ 140/90 mmHg or home blood pressure monitoring (HBPM)≥ 135/85 mmHg (Fig. 5) (141, 142). BP can be measured in the office and in ambulatory settings (ie, HBPM and ABPM). While the opportunistic screening of hypertension is typically performed using office BP measurement, the diagnosis of hypertension in most cases should be confirmed with either HBPM or ABPM (142). A recent meta-analysis showed a higher specificity for the diagnosis of hypertension with ABPM in comparison to office BP measurements (143). Both HBPM and ABPM identify white-coat and masked hypertension, but ABPM offers a more comprehensive assessment as it also evaluates nocturnal hypertension and the nocturnal dipping status. However, ABPM is more expensive and less practical than HBPM and office measurements, and may also be less available (144). The frequency of hypertension was reported to be higher in patients with acromegaly assessed by ABPM than office BP measurements, with 31% of the non-hypertensive cases by office BP measurement being identified by ABPM (145).

### Cardiomyopathy

#### Epidemiology

In acromegaly patients younger than 40 years with active disease for 3-7 years, 54% had evidence of LVH on echocardiography, with this rate rising to 72% in patients aged 41-60 years and with an estimated disease duration of 5-15 years (49, 146). Progression to systolic dysfunction occurs in <3% of acromegaly patients, and the presence of overt congestive heart failure is rare, ranging between 1% and 4% in patients with active disease (41). A recent systematic review reported a myocardial hypertrophy mean prevalence of 59% in acromegaly, while congestive heart failure was estimated at 11% (7). Hong et al (147) reported that congestive heart failure in acromegaly patients was approximately 2-fold higher than in the controls, and remained significantly higher by 1.5-fold after adjusting for diabetes, hypertension, and dyslipidemia. However, recent studies employing cardiac MRI reported a lower prevalence of myocardial hypertrophy in acromegaly, ranging from 5% to 24%. These prevalence rates contrast with the high hypertrophy prevalence described in older echocardiography-based studies (as high as 80%) (52, 62, 148). This prevalence rate discrepancy may be related to the distinct accuracy of the different methods and due to the several limitations of the 2D-echocardiography in comparison to cardiac MRI (50).

#### Screening and diagnosis

Although several consensus and position statements have addressed the management of acromegaly and its complications, a universal approach for screening of cardiomyopathy and CV disease has not yet been established. In particular, there is no general agreement on the preferred diagnostic methods or how often to test (10).

Echocardiography is widely available and has been traditionally considered the standard technique to evaluate cardiac hypertrophy. However, recent studies tend to document by MRI in order to provide accurate measurement of LVM and myocardial fibrosis in the general population (52, 149, 150). Cardiac MRI provides accurate measurements of ventricular wall thickness, myocardial mass, atrial and cardiac geometry, and it can inform about dense myocardial fibrosis. On the other hand, cardiac MRI is less susceptible to operator-related factors, errors related with calculations of ventricle mass, or artifacts related with patient's body weight and thoracic kyphosis (50, 52). Thus, cardiac MRI may be superior to echocardiography to characterize cardio-morphological alterations, which can explain the methodological shift in recent studies. However, in routine clinical practice, echocardiography is more commonly used because of the lower costs and wider availability than cardiac MRI. Thus, in general, cardiac MRI should be reserved for selected cases, only when clinically indicated.

Due to the high prevalence of cardiomyopathy, hypertension and other structural abnormalities, such as valvular heart disease in individuals with acromegaly, a baseline echocardiogram, as well as an electrocardiogram (ECG), are indicated, even if cardiac symptoms or known heart disease are absent (Table S2) (58, 151, 152). Echocardiogram and ECG can be repeated annually or biannually, depending on the presence of underlying cardiac symptoms, hypertension control, and other CV complications, as well as based on acromegaly activity and on the severity of GH excess. Cardiac exams may be performed comprehensively and more frequently in patients with severe cardiomyopathy, progressing cardiac-related symptoms, and poorly controlled disease (10). Other useful resources to diagnose and monitor cardiomyopathy besides cardiac MRI, include 24-hour Holter ECG monitoring and stress tolerance test, particularly in cases of abnormal ECG and/or echocardiogram, as well as for assessment completeness in cases with suspected heart disease or dysrhythmias (Table S2) (10, 58, 151, 152). Additional diagnostic tests may be used to identify specific CV complications and should be requested based on individual clinical indication, following the same indications as for the general population (Table S2) (58, 153, 154). Serum NT-pro-BNP may be useful for the prediction of CV events in patients with acromegaly (155). A recent study measured serum NT-pro-BNP levels in 76 patients with acromegaly and compared with other predictors of CV events, and they found that mean NT-pro-BNP concentration was higher in patients who had CV events than in those who did not, and in patients who died due to CV events than in those deceased due to other causes. They observed that the risk of developing a CV event was 19 times greater in patients with serum NT-pro-BNP levels above 91.55 pg/mL. In another study where the relationship between serum IGF-1 levels and NT-pro-BNP concentrations was investigated in the general population, it was found that higher IGF-1 levels and IGF-1/IGFBP-3 ratio were associated were higher NT-pro-BNP values in women, while a U-shaped relation of baseline IGF-1, IGFBP-3, and IGF-1/IGFBP-3 ratio with increased NT-pro-BNP levels was observed in men (156).

### Cardiac valve disease

#### Epidemiology

The prevalence of heart valve disease at the time of acromegaly diagnosis varies across studies, depending on the definition and severity of cardiac valve disease and on the population studied. Several echocardiographic studies suggested a high burden of valvular abnormalities in acromegaly, and its severity seems to correlate with duration and activity of acromegaly (Table 4) (8, 10, 39, 57, 65-68, 157-159).

**Table 4:** Prevalence and characteristics of cardiac valve disease in acromegaly

Study (Year)	Design/N	Cardiac valve disease prevalence	Valve(s) involved	Key observations
Colao et al (2003) (65)	Case-control, 42 active, 22 cured, 64 controls	86% (active), 73% (cured), 24% (controls)	Mitral and aortic; abnormalities persisted post-cure	Valve disease persists after biochemical cure; not always associated with LVH
Pereira et al (2004) (67)	Prospective, 40 acromegaly, 120 controls	22% (acromegaly) vs 6.7% (controls) significant valve disease	Aortic regurgitation (30% vs 7%), mitral regurgitation (5% vs 0%)	Risk increases with disease duration (OR 1.19/year)
van der Klaauw et al (2006) (158)	Prospective, 37 acromegaly	Baseline: 46%; Follow-up: 67%	Mitral regurgitation increased (32%→60%); aortic unchanged	Progression seen only in active disease, not controlled
Ságová et al (2023) (68)	Case-control, 129 acromegaly, 80 controls	43% (acromegaly)	Predominantly mitral regurgitation (31%)	IGF-1, BMI, lean mass correlated with LVM
Hinojosa-Amaya et al (2021) (66)	Retrospective, 110 acromegaly	87.3% any valve defect; 14.6% significant	Not specified	Concentric LV remodeling common; early diagnosis recommended
Abreu-Lomba et al (2025) (159)	Retrospective, 193 acromegaly	6.6% isolated cardiac valve disease; 6.6% LVH + cardiac valve disease	Not specified	AHD defined as structural/functional abnormality; carpal tunnel syndrome associated

Abbreviations: AHD: acromegalic heart disease; BMI: body mass index; GH: growth hormone; IGF-1: insulin-like growth factor 1; LV: left ventricle; LVH: left ventricular hypertrophy. LVM: left ventricular mass; OR: odds ratio.

In a case-control study of 42 patients with active acromegaly, the prevalence of any cardiac valve abnormality (including mild lesions) was 86% at diagnosis compared to 24% in matched controls; this prevalence remained elevated at 73% even in patients with cured acromegaly, compared to 9% in matched controls (65). A large study found valve defects in 87.3% of patients at baseline echocardiogram, with 14.6% having significant valvular heart disease (66). Another prospective study reported significant heart valve disease in 22% of acromegaly patients at diagnosis, with aortic valve regurgitation in 30% and moderate or greater mitral regurgitation in 5% of patients (67). Additional data from a multicenter study identified valvulopathies in 6.6% of patients with acromegaly, but this low rate likely reflects only clinically significant cases (159). In a recent case–control study valvulopathy was reported in 43% of acromegaly patients, with mitral regurgitation being the most common lesion (31%) (68). In summary, the prevalence of any echocardiographically detectable heart valve abnormality in newly diagnosed acromegaly is up to 87%, while the prevalence of moderate or severe valvular heart disease is lower at 14-22%. The most frequently affected valves are the mitral and aortic valves, with regurgitation being the predominant abnormality (Table 4) (67, 68).

The risk of valvular disease increases with longer disease duration, advancing age, and in patients with poorly controlled GH excess. The odds of valvular disease increase by 19% per year of exposure to elevated GH levels (66, 67, 159). Moreover, valvular disease is only partially reversible with control of acromegaly, as valve fibrosis may persist after normalization of GH/IGF-1 levels (65, 67).

#### Screening and diagnosis

Valvular heart disease, particularly mitral and aortic regurgitation, is often subclinical and becomes more prevalent with longer disease duration and inadequate biochemical control, contributing to heart failure and increased CV morbidity (1, 65-68, 159). Therefore, screening for cardiac valve disease with transthoracic echocardiography is recommended at the diagnosis of acromegaly and may be needed periodically during the follow-up (Table S2) (58). However, clinical examinations should precede the conduction of serial cardiac ultrasound examinations.

Echocardiographic studies demonstrated that up to 86% of patients with active acromegaly and 73% of those with controlled disease have some form of mitral or aortic valve abnormality, with significant regurgitation present in a minority of patients (68). Advanced echocardiographic techniques, such as speckle tracking echocardiography, may detect subclinical myocardial and valvular dysfunction before overt changes in ejection fraction or clinical symptoms, supporting their use in selected cases for early risk stratification (160). Additional CV risk factors should be aggressively managed, as they accelerate the progression of cardiac complications (159, 161).

### Cardiac arrhythmias and conduction defects

#### Epidemiology

Acromegaly is associated with an increased prevalence and incidence of cardiac arrhythmias, particularly atrial fibrillation and complex ventricular arrhythmias (9). Dysrhythmias may be detected in up to 48% of patients with acromegaly, and as high as 90% of patients with active disease (70, 162, 163). However, data from a recent systematic review reported a lower mean prevalence of 13% (7). Several studies showed that patients with acromegaly have a higher prevalence of ECG changes compared to controls that increase the risk for ventricular arrhythmia, such as prolongation of the QT interval, QT dispersion, or changes in Tp-e/QT ratios (164, 165).

Kahaly et al (74) found that 48% of individuals with acromegaly exhibited complex ventricular arrhythmias, compared to only 12% in controls. In that study, the frequency of premature ventricular complexes increased with the duration of acromegaly, and the severity of arrhythmia correlated with LVM but not with GH levels (74). Similarly, in a large matched cohort study comprising 1874 acromegaly patients and 9370 controls, the risk of atrial fibrillation was higher in the acromegaly group (HR 1.59; 95% CI 1.09-2.31), even after adjustment for major cardiometabolic risk factors (147). Overall, atrial fibrillation seems to be more common than ventricular arrhythmias. For example, in the French study with 3551 patients included, the prevalence of atrial fibrillation was 7.7%, while ventricular tachycardia/fibrillation was present in only 0.8% of patients (37). In addition, late potentials (recognized predictors of malignant ventricular arrhythmias and sudden cardiac death) are commonly detected in this population (6). This is clinically relevant, as arrhythmias are closely linked to increased mortality, mainly through its association with sudden cardiac death. In the Dutta study, 6 out of 150 acromegaly patients had overt congestive heart failure (4.0%), and 3 of these 6 patients died due to refractory ventricular arrhythmias (166). However, it should be taking into account that the 6 patients had advanced CV disease as 4 patients had New York Heart Association Functional Classification (NYHA) class IV dyspnea and 2 had class III and left ventricular ejection fraction was 30% or less with echocardiographic features suggestive of global biventricular hypokinesia in all patients. Table 5 summarizes the cardiac arrhythmias described in patients with acromegaly and their prevalence.

**Table 5:** Prevalence of cardiac arrhythmias in patients with acromegaly

Study, year	Study population (N)	Overall prevalence	Type of arrhythmias
**Hayward et al** 1987 (163) (ECG diagnosis)	256	3.5%	Repolarization disorders or intraventricular conduction defects
**Rodrigues EA** (20)**, 1989** (ECG diagnosis)	34	41%	Conduction disorders
**Kahaly G** (74)**, 1992** (ECG diagnosis)	32	48%	Complex ventricular arrhythmias (Lown III-IV) in 48% and repetitive ventricular arrhythmias (Lown IV a/b) in 31%
**Hermann** (167), **2001** (ECG diagnosis)	48	56%	Late potentials in ECG
**Lombardi** (168)**, 2002** (24-hour dynamic ECG diagnosis)	19	40%	16.6% had supraventricular premature beats, and 35% ventricular premature beats
**Warszawski et al** (162) 2016 (Diagnosis with 24h-Holter)	36	89%	Isolated and paired ventricular ectopy in 71% and 21%, isolated and paired supraventricular ectopy in 89% and 39%, non-sustained ventricular tachycardia in 11% and non-sustained supraventricular tachycardia in 46%
**Melkozerov** (169)**, 2020** (ECG diagnosis)	461	42%	Most frequent: bradycardia 19.1% and conduction disorders of bundle branch blocks 14.5%.
**Hong S** (147), 2022 (ECG diagnosis)	1874	2.24% (incidence)	Atrial fibrillation in 2.24% (vs 1.25% in controls)

Abbreviation: ECG: electrocardiogram

#### Screening and diagnosis

Considering the high prevalence of conduction abnormalities and arrhythmias in patients with acromegaly, at least one ECG should be performed at the time of the diagnosis of acromegaly (10, 58) (Table S2). The 24-h Holter ECG monitoring is advised if there is an abnormal baseline ECG and clinical suspicion of cardiac dysrhythmia (170). Nevertheless, the last Acroconsensus on the management of acromegaly-associated comorbidities only recommends ECG, as arrhythmias are relatively uncommon in acromegaly, and when present, these are likely related to structural heart disease, particularly cardiomyopathy (141).

### Coronary artery disease

#### Epidemiology

The prevalence CAD in acromegaly has been a matter of controversy and seems to be lower than expected. A recent review reported a lower mean prevalence of CAD in acromegaly at 6% (7). Paisley et al (171). did not report an increased prevalence of CAD, nor carotid atherosclerosis, or increased carotid intima media thickness in acromegaly patients when compared to control subjects. Data from Italy, Belgium, France, and Mexico suggest that CAD rates are similar to those of the general population (10, 31, 172, 173). Moreover, the incidence of myocardial infarction or other CV events in adequately treated acromegaly patients does not differ from the general population (81, 147), nor from patients with non-functioning pituitary tumors (174). Concomitant presence of additional CV risk factors such as smoking, SAS, hyperglycemia, dyslipidemia, and obesity, seems to have a greater impact on the development of CAD and atherosclerosis, rather than the long-term GH and IGF-1 excess (10, 175). Other studies showed an increased CAD risk in patients with acromegaly (27, 172, 176), and elevated IGF-1 has been associated with an increased risk of mortality, diabetes, major adverse cardiovascular events and cancer (176).

Studies using non-invasive imaging techniques (eg, coronary computed tomography [CT] or calcium scoring) have yielded conflicting results, with some showing increased coronary artery calcium (177), while others reporting preserved coronary flow reserve (178), suggesting that other mechanisms beyond atherosclerotic disease may drive CV morbidity in these patients.

#### Screening and diagnosis

Given the inconsistent and relatively uncommon association between acromegaly and CAD, the routine screening for CAD in asymptomatic acromegaly patients is not recommended by current guidelines. Nevertheless, traditional CV risk factors, such as age, hypertension, dyslipidemia, insulin resistance, smoking, and SAS, remain highly prevalent in acromegaly and should be systematically evaluated, and baseline CV screening should be performed (Table S2) (58, 141).

Specific investigation for CAD in acromegaly patients should follow the general population recommendations and should be guided by the CAD risk stratification and clinical manifestations (179). If ischemic heart disease is suspected, different investigations can be requested based on local resources and experience (eg, stress tolerance test, exercise treadmill, CT calcium score) (Table S2) (58).

### Cerebrovascular disease

#### Epidemiology

Several cohort and registry studies demonstrated a 2-fold to 3-fold increased risk of cerebrovascular disease in acromegaly than in the general population (22, 33). However, recent series suggested that the incidence of stroke in acromegaly patients with controlled disease does not differ from the general population (81), nor from patients with non-functioning pituitary tumors (81). Specifically, the German Acromegaly Registry (81) reported an incidence of stroke similar to matched controls (standardized incidence ratio 1.17), similar to the Danish nationwide acromegaly cohort (23). However, the risk for stroke is notably higher in the presence of additional CV risk factors (13), as well as in irradiated patients (180, 181), however, studies applying newer stereotactic techniques reported low stroke risk, with the highest quality nationwide study identifying no significant difference in ischemic stroke (182).

Cerebrovascular disease accounts for approximately 5-10% of mortality in older series, although its incidence has declined over the recent decades, likely due to earlier diagnosis, improved disease control, and reduced use of radiotherapy (7, 23, 183). A meta-analysis confirmed this decrease in mortality rates, including 26 observational studies (26), which showed that cerebrovascular mortality was higher in studies published before 2008 (SMR of 3.4) but declined to near-normal levels after 2008 (SMR 1.3). The risk of cerebrovascular death is greatest in patients with prolonged diagnostic delay or persistent active disease (184-186).

A special risk factor for hemorrhagic cerebrovascular events are the cerebral aneurysms, which appear to be more prevalent in patients with acromegaly than in the general population. In a large Japanese cohort (187), cerebral aneurysms were found in 4.3% of the 208 patients with acromegaly, compared to 1.8% of the 7390 control subjects. In contrast, Manara et al (90). reported a considerably higher prevalence of 17.3% among 152 acromegaly patients evaluated with MR angiography. Given the 3.2% prevalence rate reported in the general population (188), acromegaly appears to confer an increased risk of cerebral aneurysm formation. However, no systematic data is available on aneurysm-specific mortality in acromegaly.

In relation to ischemic stroke, one recognized risk factor in patients with acromegaly is diabetes mellitus (37). In the French cohort study of 3551 acromegaly cases, it was found that those patients with diabetes were at higher risk for most comorbidities: myocardial infarction (odds ratio [OR] 3.14 [1.92-5.13]), ischemic stroke (OR 1.64 [1.18-2.28]), and cancer of any type (OR 1.53 [1.27-1.84]) (37). Moreover, other cardiometabolic disorders like hypertension and dyslipidemia are higher in acromegaly patients than in general. These predisposing factors are concerned with the induction of acute ischemic stroke. High circulating GH levels in acromegaly may predispose to the development of acute ischemic stroke (189).

#### Screening and diagnosis

Since the current evidence does not suggest an increased risk of cerebrovascular disease in acromegaly, no specific screening program is recommended (Tables S3 and S4) (58, 190). Hence, screening tools and timing should be performed based on the patient's CV risk profile (Framingham score (191)), as for the general population. Brain imaging studies should be performed as clinically indicated, including in the acute setting if a stroke is suspected. An overview of the different diagnostic tools and their indication is provided in Table S3 and S4 (58).

### Thrombosis and hypercoagulability

#### Epidemiology

Isolated cases of venous thromboembolism (192), massive pulmonary embolism (192, 193), and intracardiac thrombus (194) have been reported in patients with acromegaly without other risk factors. Nevertheless, acromegaly is not among the classically high-risk comorbidities associated with this condition. Most of the available nationwide cohorts do not mention thrombotic events in acromegaly, either at diagnosis or during the follow-up (123, 128, 130, 173, 195). Only the Danish national registry reported an increase in venous thromboembolism, with an adjusted HR of 2.3 (95% CI: 1.1-5.0) and 9.3 (95% CI: 5.4-16.0) at diagnosis and during the 30-year period after, respectively (23). However, it has been hypothesized that a hypercoagulable state may be fostered in acromegaly, which could be a relevant factor for atherothrombotic complications (26).

#### Screening and diagnosis

Routine screening for thrombosis or laboratory markers of hypercoagulability is not currently recommended in patients with acromegaly, either at diagnosis or during follow-up. Although acromegaly may be associated with a hypercoagulable state, current evidence is insufficient to justify routine screening for thrombophilia or subclinical thrombosis in asymptomatic patients, or, for instance, to recommend thromboprophylaxis in acromegaly. Targeted coagulation tests may be appropriate in selected cases, particularly in individuals with a personal or family history of thrombosis, unexplained vascular events, or additional predisposing risk factors, as for the general population recommendations (196).

### Diabetes Mellitus and impaired glucose metabolism

#### Epidemiology

Diabetes mellitus and glucose metabolism disorders are prominent comorbidities in acromegaly, affecting up to two-thirds of newly diagnosed acromegaly patients (7). The prevalence of diabetes mellitus increases with duration of acromegaly (33) and, notably, diabetes frequently persists despite biochemical remission of acromegaly (48). A systematic review (3) revealed a pooled diabetes prevalence of 28-33% across continents, but single-center and regional cohorts reported higher glucose abnormality rates as high as 52% (197) and over 60% (198). In a Spanish study performed in 2004, including 1219 cases, diabetes mellitus was reported in 37% (195), while in a more recent series, including more than 600 patients, the diabetes prevalence was 26% in GH-secreting pituitary adenomas and 13% in GH&PRL co-secreting tumors (121). In two distinct Italian series, the prevalence of diabetes mellitus was lower at 14% and 16% (199, 200), whereas the French acromegaly registry reported a prevalence rate of 47% and 27% (Table 6).

**Table 6:** Prevalence of diabetes mellitus in the main registry series of patients with acromegaly

Author, year [reference]	Country	Study population Size (n)	Prevalence of diabetes (%)
**Gatto et al 2018** (199)	Italy	74	14.6%
**Arosio M. 2012** (200)	Italy	1512	16%
**Vallette S, 2013** (124)	Canada	649	27%
**Schöfl C, 2013** (125)	Germany	1344	45.0%
**Kwon O, 2013** (126)	South Korea	1350	28.6%
**Howlett TA 2013** (127)	UK	2572	28%
**Portocarrero-Ortiz LA, 2016** (128)	Mexico	2057	18.4%
**Vila G, 2016** (129)	Austria	607	29%
**Fieffe S, 2011** (201)	France	519	47.5%
**Maione L, 2017** (130)	France	980	27%
**Esposito D, 2018** (25)	Sweden	1089	27%
**Mestron A, 2004** (195)	Spain	1219	37%
**Araujo-Castro M, 2024** (121)	Spain	604	26%

#### Screening and diagnosis

The current acromegaly guidelines strongly recommend systematic screening for diabetes and glucose intolerance at diagnosis and during follow-up (1, 141, 202). Initial screening should include measurement of FPG, HbA1c, and OGTT. After the diagnosis of acromegaly, FPG, and HbA1c should be performed every 6 months, especially those with CV risk factors, and following changes in the medication or disease activity. The most-accepted diagnostic criteria for diabetes and glucose intolerance in acromegaly align with the American Diabetes Association and the World Health Organization guidelines: FPG ≥126 mg/dL, 2-hour OGTT glucose ≥200 mg/dL, or HbA1c ≥6.5% (203).

In some specific situations, it is recommended to re-screen the patients for glucose metabolism abnormalities, particularly after initiation of SRLs (especially pasireotide), dose escalation, or therapy switching. Pasireotide, in particular, necessitates close glycemic monitoring in the short term due to the high risk of early therapy-induced hyperglycemia, which may appear in approximately 50% of patients treated with pasireotide (204, 205).

### Dyslipidemia

#### Epidemiology

Dyslipidemia is highly prevalent in acromegaly, with up to 61% of patients exhibiting lipid abnormalities regardless of their GH and IGF-1 levels (9, 108, 157, 206) (Table 7). Typical lipid profile changes in acromegaly include elevated total cholesterol, LDL, VLDL, triglycerides, and Lp(a), along with decreased HDL cholesterol (9, 108, 157, 206) (107).

**Table 7:** Prevalence of dyslipidemia in the main registry series of patients with acromegaly

Author, year [reference]	Country	Study population Size (n)	Prevalence of dyslipidemia (%)
**Gatto F, 2018** (199)	Italy	74	NR
**Arosio M. 2012** (200)	Italy	1512	NR
**Vallette S, 2013** (124)	Canada	649	NR
**Schöfl C, 2013** (125)	Germany	1344	NR
**Kwon O, 2013** (126)	South Korea	1350	NR
**Howlett TA 2013** (127)	UK	2572	NR
**Portocarrero-Ortiz LA, 2016** (128)	Mexico	2057	24%
**Vila G, 2016** (129)	Austria	607	NR
**Fieffe S, 2011** (201)	France	519	NR
**Maione L, 2017** (130)	France	980	NR
**Esposito D, 2018** (25)	Sweden	1089	NR
**Mestron A, 2004** (195)	Spain	1219	NR
**Pascual-Corrales E, 2024** (103)	Spain	604	29.2%

Hypertriglyceridemia affects 33-40% of acromegaly patients, roughly 3 times higher than in the general population, while low HDL levels are observed in 39-47% of patients (108). Furthermore, an increased proportion of small, dense LDL particles, which are more atherogenic, is commonly observed in patients with acromegaly (207). Other apolipoproteins, such as Apo A-I and Apo E, involved in lipid transport, are also found elevated in these patients (108).

#### Screening and diagnosis

Given the absence of acromegaly-specific recommendations for lipid screening and the heterogeneity of reported findings, current practice is to follow the guidelines for the general population (208). Nonetheless, in patients with additional risk factors (eg, diabetes, obesity, or previous radiotherapy), a more proactive approach to lipid assessment and CV risk stratification may be warranted. Future studies are needed to define lipid-related risk in this population better and to determine whether tailored screening or treatment algorithms would provide incremental benefit (209).

### Sleep apnea syndrome

#### Epidemiology

In patients with acromegaly, respiratory complications frequently present as respiratory failure and obstructive SAS (70). Obstructive SAS is defined by an apnea–hypopnea index greater than 5 events per hour. This condition is associated with sleepiness, fatigue, poor concentration, and increased mortality and morbidity linked to CV disease (49). In this regard, it should be noted that respiratory disorders constitute a relevant cause of illness and impaired physical performance in patients with acromegaly. In relation to respiratory mortality, a recent retrospective analysis from the UK Acromegaly Register reported an excess mortality due to vascular and respiratory disease in patients with acromegaly and the risk was highest in the first 5 years following diagnosis, but was mitigated by normalizing GH levels (27).

It is estimated that 20% to 80% of patients with acromegaly are affected by sleep apnea, mainly due to the obstructive form (two-thirds of cases) (70, 210, 211). Although it may be expected a higher prevalence in patients with active disease, a meta-analysis of 21 articles for a total of 24 studies with 734 patients included that compared obstructive SAS prevalence in patients with active and controlled acromegaly found a similar prevalence of obstructive SAS between groups (ES = −0.16; 95% CI, −0.47 to 0.15; *P* = .32) (212). On the other hand, the prevalence of acromegaly in patients with obstructive SAS ranges between 0.14% and 0.35%, being higher than the reported the general population (0.003%–0.014%) (213-215). The Danish national registry reported an HR of obstructive SAS of 11.7 (95% CI, 7.0 to 19.4) in acromegaly compared with the general population (23). However, guidelines on SAS management do not propose systematic screening for the presence of acromegaly (216).

#### Screening and diagnosis

Guidelines for diagnosis and treatment of acromegaly suggest a screening test and IGF-1 measurement when there are multiple associated comorbidities, including SAS (217). In those patients with a newly diagnosed acromegaly, clinical guidelines recommend that every patient should have a careful symptomatic assessment (eg, by Epworth score), and if necessary laboratory assessment, for sleep apnea at the time of diagnosis, in collaboration with a respiratory physician (152). The gold standard for the diagnosis of SAS is polysomnography. Furthermore, it should be taking into account that sleep apnea does not consistently resolve after effective treatment of acromegaly, so post-treatment evaluation is recommended (218).

## Recommendations for the diagnosis and screening of cardiovascular comorbidities in acromegaly

Currently, there are no guidelines specifically focused on the diagnosis and screening of CV comorbidities in acromegaly; however, a few consensus recommendations have been generated (219, 220). The management of diabetes and dyslipidemia should follow general population guidelines (Table 8).

**Table 8:** Evidence robustness assessment for cardiovascular complications in acromegaly

CV complication	Level of evidence	Number of studies	Total patients studied	Key study types	Evidence quality	Main limitations
**Hypertension**	High	>50 studies	>20 000	Large registries, meta-analyses, prospective cohorts	★★★★★	Variability in diagnosis methods (office vs ABPM); heterogeneous definitions across studies
**Cardiomyopathy/LVH**	High	>40 studies	>5000	Echocardiography studies, cardiac MRI studies, prospective cohorts	★★★★★	Different imaging modalities yield different prevalence; older echo-based studies overestimate compared to MRI
**Valvular Heart disease**	Moderate-High	∼15 studies	∼1500	Case-control studies, prospective echocardiography series	★★★★⋆	Most cases are mild; clinical significance unclear; limited long-term outcome data
**Cardiac arrhythmias**	Moderate	∼10-15 studies	∼1000	ECG studies, Holter monitoring, registry data	★★★⋆⋆	Small cohorts; heterogeneous definitions; limited data on clinical outcomes and sudden death
**Diabetes Mellitus**	High	>30 studies	>15 000	Large registries (n = 10), meta-analyses, prospective cohorts	★★★★★	Variability in diagnostic criteria (FPG vs OGTT vs HbA1c); different study designs
**Coronary artery disease**	Moderate	∼15-20 studies	∼2000	Imaging studies (CT, angiography), registry data	★★★⋆⋆	Conflicting results; unclear if prevalence truly elevated; heterogeneous patient populations; limited prospective data
**Cerebrovascular Disease**	Moderate	∼10 studies	∼5000	Registry data, national cohorts	★★★⋆⋆	Most data from registries; limited mechanistic studies; confounding by radiotherapy in older cohorts
**Hypercoagulability**	Low	∼8-10 studies	<300	Small case-control studies	★★⋆⋆⋆	Very small cohorts; inconsistent findings; most studies <50 patients; lack of prospective data
**Thrombotic events**	Low	5-7 studies	∼2000	Registry data, case reports	★⋆⋆⋆⋆	Mostly registry mentions; very few dedicated studies; incidence unclear; limited mechanistic understanding
**Dyslipidemia**	Moderate	∼15 studies	∼1500	Cross-sectional studies, small cohorts	★★★⋆⋆	Prevalence varies widely (33-61%); limited data on clinical impact; unclear relationship with atherosclerosis in acromegaly
**Sleep apnea syndrome**	Moderate-High	∼20-25 studies	∼2500	Polysomnography studies, meta-analyses	★★★★⋆	Variable diagnostic criteria; unclear reversibility with treatment; limited prospective data
**Overall mortality**	High	>40 studies	>25 000	Meta-analyses, national registries, long-term cohorts	★★★★★	Temporal trends complicate interpretation; changing treatment paradigms; residual confounding

**Evidence Quality Rating Scale:**
 ★★★★★ **Very high**: Multiple large studies (>1000 patients), consistent findings, meta-analyses available, prospective data ★★★★⋆ **High**: Several studies (>500 patients), mostly consistent findings, some prospective data ★★★⋆⋆ **Moderate**: Multiple small-moderate studies (100-500 patients), some inconsistency, mostly retrospective ★★⋆⋆⋆ **Low**: Few small studies (<100 patients), significant inconsistency, case series predominate ★⋆⋆⋆⋆ **Very low**: Very few studies, case reports/small series only, highly inconsistent or absent data

**Summary of Evidence Gaps:**

**Well-established (high confidence):**
 Hypertension: ∼35-45% prevalence, clear pathophysiology, treatment benefits established Cardiomyopathy: ∼15-60% prevalence (method-dependent), reversible with treatment, clear mechanisms Diabetes: ∼20-45% prevalence, well-defined risk factors, treatment effects known Mortality: Clear historical excess, improvement with modern treatment, robust registry data

**Moderately established (moderate confidence):**
 Valvular disease: Prevalence clear but clinical significance uncertain Arrhythmias: Present but relationship to disease activity unclear CAD: Paradoxically lower than expected; mechanisms unclear Cerebrovascular disease: Risk elevated but magnitude uncertain Sleep apnea: Common but reversibility uncertain

**Poorly established (low confidence):**
 Hypercoagulability: Laboratory abnormalities inconsistent Thrombotic events: Clinical significance unclear despite laboratory changes Precise mechanisms linking GH/IGF-1 excess to specific complications remain incomplete

**Abbreviations:** ABPM, ambulatory blood pressure monitoring; CAD, coronary artery disease; CT, computed tomography; ECG, electrocardiography; FPG, fasting plasma glucose; GH, growth hormone; HbA1c, glycated hemoglobin; IGF-1, insulin-like growth factor 1; LVH, left ventricular hypertrophy; MRI, magnetic resonance imaging; OGTT, oral glucose tolerance test.

There are many exams available to diagnose these comorbidities (Tables S2 and S3) (58). The method of choice and its frequency is not established; thus, it may be decided on an individual basis considering the underlying CV risk factors, cardiometabolic comorbidities, and duration and severity of the disease (10, 57).

Given the high prevalence of CV disease in acromegaly, which may be further aggravated in hypertensive patients, a general cardiac status assessment at diagnosis and during follow-up should be performed. The minimal baseline assessment should include BP measurement (at least every 6 months), an ECG (annually), and an echocardiogram (annually, particularly in case of hypertension, cardiac abnormality, active disease, or moderate–high CV risk) (152). The use of echocardiograms at diagnosis and during follow-up for all patients is recommended, especially for patients with hypertension and/or diabetes mellitus (151). In the presence of clinical disease or suspicion of it, or before pituitary surgery, a detailed cardiac evaluation is advisable.

Regarding CAD and atherosclerosis, due to the heterogeneity in prevalence across different studies, establishing an optimal screening approach is complex. The current evidence does not seem to suggest an increased risk of CAD in acromegaly. Hence, screening tools and timing should be performed based on the patient's CV risk profile (Framingham score), as for the general population. In case of suspected ischemic heart disease, the assessment includes an ECG or a stress and/or exercise tolerance test to evaluate cardiac performance, while other exams, such as CT angiography, CT calcium score, coronary catheterization, positron-emitted tomography, and carotid ultrasonography, allow a better evaluation of arterial damage and provide further information when needed (10, 57, 151).

Despite the diagnostic approach followed to identify CV comorbidities, it is important to maintain a longitudinal monitoring of the cardiometabolic status of these patients, and a rigorous management of their individual complications during the long-term follow-up (10, 57). In addition, for those patients with symptomatic CV disease we recommend referring these patients to the dedicated specialist who will decide about the optimal management.

## Impact of acromegaly treatments on cardiovascular and metabolic comorbidities

### Effects on hypertension

There is an inverse relationship between serum IGF-1 and BP in conditions associated with low IGF-1 concentrations, and a positive correlation between IGF-1 and BP in high IGF-1 conditions (208). Addressing GH hypersecretion in acromegaly is crucial, as reducing or normalizing GH/IGF-1 levels leads to BP reduction. Surgery is the preferred first-line treatment for acromegaly, as it offers the potential for cure (1, 221, 222). Following successful surgery, most patients have BP improvements, with greater benefits in those with pre-existing hypertension (122, 223). In a recent large Iberian acromegaly cohort study, it was observed a significant BP improvement just 3 months after the pituitary surgery with a marked reduction of systolic and diastolic BP. Hypertension remission was achieved in around 15% of hypertensive acromegaly patients after pituitary surgery, and it was more likely in patients who had a greater decrease in GH and IGF-1 levels after the operation (122).

Several other studies have demonstrated that GH/IGF-1 normalization, regardless of the treatment modality, positively impacts BP reduction, particularly in hypertensive patients (11-13). Treatment with first-generation SRLs (fgSRLs) (16) and pegvisomant (17) reduces the progression of LVH and improves structural cardiac dysfunction, including left ventricular ejection fraction. The effects of pasireotide in BP and hypertension remain to be evaluated; however, its modulatory effects on other relevant CV parameters are relevant. Pasireotide increases FPG and leads to a rise in diabetes prevalence, but on the other hand, improves the lipid profile by increasing HDL cholesterol and decreasing triglyceride levels (224-227). Clinically non-relevant QT interval prolongations have also been reported with pasireotide (228).

Given the pathophysiology of hypertension in acromegaly, it is not surprising that the prevalence is significantly higher in biochemically uncontrolled patients (172, 229). This was observed in a retrospective observational multicenter study evaluating BP changes in 105 hypertensive acromegalic patients at diagnosis and after 24 months (230). In this study, no difference was found in GH/IGF-1 levels between patients with mild or severe hypertension. While diastolic BP and left ventricular mass index improved in all patients under acromegaly treatment, systolic BP and cardiac function improved only in the patients who achieved biochemical control. Moreover, it was noted that uncontrolled acromegaly patients required significantly more antihypertensive medication (230).

Several studies evaluated the effect of different GH/IGF-1-lowering treatments on BP and CV morbidity. Surgical intervention has been shown to have the fastest effect, improving hypertension within 3 to 6 months and restoring a normal circadian BP rhythm (15, 231, 232). Significant improvements in both systolic BP (15, 231, 232) and diastolic BP (16, 233) have been described following adequate treatment of acromegaly.

A meta-analysis dated from 2007 showed no significant decrease in BP in patients under fgSRL treatment (16). This contrasts with data from two prospective studies where a reduction in BP in patients treated for at least 12 months has been shown (234, 235). While some studies describe a decrease in systolic BP under combined treatment (236, 237), other studies reported an effect only in biochemically controlled patients (229) or no effect (12). The importance of biochemical control was also shown in a retrospective study including 30 hypertensive and 28 normotensive patients with acromegaly. Biochemical control of acromegaly after 24 months with fgSRLs in monotherapy or in combination with pegvisomant had a beneficial effect on BP regulation (135). In addition, less progression of LVH and improved markers of structural cardiac dysfunction were observed in biochemically controlled patients in other studies (16, 238). Overall, the positive effect of fgSRLs and pegvisomant on BP appears to be dependent on long-term control of acromegaly. There is no data on the impact of cabergoline or radiotherapy on BP values in acromegalic patients. However, a recent study described a weight and BMI reduction in acromegaly patients treated with cabergoline1. Although BP was not studied, the weight loss related to the use of cabergoline in acromegaly may, in theory, improve the BP.

The impact of hypertension on clinical outcomes was demonstrated in the ACROSTUDY involving 2090 patients with acromegaly, 1344 of whom had hypertension. Although the mortality rate did not differ from the general population with hypertension (239), patients with hypertension had a 3.3-fold increased mortality rate compared to the normotensive patients (240).

### Effects on cardiovascular complications

Treatment with fgSRLs can reduce cardiac dysrhythmia in some cases by reducing heart rate, premature ventricular beats, and QT interval (241, 242). Pegvisomant reduces the mean heart rate and reduces the risk of arrhythmias. In the Auriemma et al study, pegvisomant reduced the prevalence of arrhythmias from 15 down to 7.7%, and its complete disappearance was observed in one patient after 18 months of pegvisomant treatment (243). Pasireotide is associated with QT prolongation (2). In relation to cabergoline, the CV effects of this drug on acromegaly remain unclear, as it may be dose and duration dependent. Maione et al (244) compared patients treated with a median cumulative dose of 203 mg of cabergoline during 35 months vs patients never treated with cabergoline and found no differences in the incidence of new valve regurgitation (40.0 vs 45.8%, respectively) and disease control. Some studies did not observe changes in the frequency of ventricular arrhythmias after successful treatment of acromegaly (245).

All stages of acromegaly-related cardiomyopathy and cardiac morpho-functional parameters can improve during successful treatment of acromegaly, although more significant improvements are seen in earlier disease stages. Conversely, the cardiac output of patients with congestive heart failure may benefit from effective acromegaly treatment, particularly their systolic function. Still, full recovery is not possible, and long-term prognosis remains poor (16, 246). The cardiac improvements following GH/IGF-1 normalization can be observed regardless of the type of acromegaly treatment (14-17). Combined therapy with pegvisomant and fgSRLs may also improve the cardiac structure and function, notably the diastolic dysfunction, in acromegaly patients resistant to isolated therapy with fgSRLs (237). In order to achieve the maximal improvement of the acromegalic cardiomyopathy, GH/IGF-1-lowering treatments must be combined with efforts and therapies to optimize other coexisting CV risk factors (50). Popielarz-Grygalewicz et al (247) suggested that fgSRLs may restore disturbed coronary function in patients with acromegaly.

### Effects on diabetes

The different medical therapies for acromegaly may impact the glycemic status variably. FgSRLs (octreotide, lanreotide), despite improving disease control, can mildly reduce insulin secretion, leading to a slight increase in HbA1c and postprandial glucose levels, without significantly affecting FPG levels (248). Those patients with pre-existing impaired glucose tolerance or diabetes are more prone to glycemic deterioration during treatment with fgSRLs (249). In contrast, pasireotide has a reversible negative impact on glycemic control due to its high potency in suppressing endogenous insulin secretion, resulting in increased FPG, HbA1c, and diabetes (250). A recent meta-analysis with 20 real-world studies comprising 409 patients, reported a significant increase in FPG (SMD − 0.8 mg/dL [95% CI: −1.0 to −0.5, *P* < .01]), HbA1c (SMD − 0.5% [95% CI: −0.7 to −0.2]) and type 2 diabetes prevalence (SMD − 11.5% (95% CI: −17.5 to −5.5) after pasireotide initiation (250). Similar results were reported from another recent meta-analysis describing a higher frequency of diabetes after pasireotide treatment (OR 3.7, 95% CI 2.9-4.7) (205). According to the data from the main clinical trials, the rates of hyperglycemia and diabetes with pasireotide treatment were approximately 41.5-42.4% and 23.6-24.2% respectively (251, 252). The hyperglycemic effect of pasireotide is mainly attributed to an impairment in insulin secretion, while insulin sensitivity remains unaffected (253). The main risk factors of hyperglycemia with pasireotide are older age, the presence of previous impaired glucose tolerance, elevated HbA1c, a history of dyslipidemia or hypertension, and BMI ≥ 30 kg/m^2^ (254). These glucose disturbances typically appear shortly after pasireotide initiation and tend to improve once the therapy is withdrawn (254, 255).

Pegvisomant has a favorable effect on carbohydrate metabolism by decreasing FPG and HbA1c, as well as improving insulin sensitivity (256-258). A meta-analysis of 30 studies with pegvisomant in monotherapy and 5 with pegvisomant in combination with fgSRLs showed a significantly decrease of FPG [effect size (ES) −0.80 mmol/L (95% CI, −1.06 to −0.55); *P* = .000], of HbA1c [ES −0.43% (95% CI, −0.56 to −0.31); *P* = .000], of fasting plasma insulin [ES −5.31 mU/L (95% CI, −10.23 to −0.39); *P* = .034], and of HOMA-IR [ES −0.61 (95% CI, −1.17 to −0.04); *P* = .034] (256). It should be noted that the combination of pegvisomant with fgSRLs mitigates the negative impact of fgSRLs on glucose metabolism, producing an overall neutral effect (256).

Dopamine agonists seem to improve insulin sensitivity and may exert a positive effect on carbohydrate metabolism (259). A study of 12 patients with acromegaly treated with bromocriptine showed a significant decrease in basal and glucose-stimulated insulin levels in all patients after treatment (260). The addition of cabergoline to pegvisomant decreases the postprandial glucose rise (261). However, a recent study aimed to evaluate the effect of cabergoline on weight and glucose metabolism in patients with acromegaly, failed to demonstrate a marked benefit on glucose metabolism (262).

### Effects on dyslipidemia

Achieving biochemical control of acromegaly positively affects cardiometabolic parameters, including the lipid profile (108). Transsphenoidal surgery has been associated with improvements in lipid levels, including reduction of triglyceride levels and an increase in HDL cholesterol levels following normalization of serum GH and IGF-1 (263). FgSRLs, particularly octreotide long-acting release demonstrated significant reductions in triglycerides, total cholesterol/HDL ratio, and Lp(a), and led to an increase in HDL cholesterol levels (264). Similarly, lanreotide has been shown to lower triglycerides and increase HDL cholesterol in non-diabetic acromegaly patients (264). However, pegvisomant has mixed effects on lipids, as it reduces Lp(a) levels but may increase total cholesterol and triglycerides in some cases (265). Pasireotide has been studied mostly in healthy volunteers, showing dose-dependent reductions in serum cholesterol and triglycerides (108). Pasireotide also seems to have a beneficial effect on the lipid profile of patients with Cushing's disease (266), but evidence concerning acromegaly is limited.

### Effect on sleep apnea syndrome

Overall, the treatment of acromegaly improves SAS in a substantial number of patients, but biochemical control does not reliably predict remission of SAS. Some studies showed that 40% of patients with controlled acromegaly have persistent SAS after treatment (118, 120, 267). In an Italian study including 36 acromegaly patients (18 active and 18 controlled), the prevalence of SAS was 56% in the active group and 39% in the controlled one, and in the longitudinal evaluation available in 6 patients, 5 showed improvement of SAS, but none recovered (118). A recent prospective study with 35 patients found that after surgery (with a remission rate of 91.4%), 20 of the 24 patients diagnosed with obstructive SAS preoperatively had an respiratory event index <5/hour postoperatively (267). SRLs may have a positive impact on obstructive SAS (118, 268, 269). Herrmann e*t al* described a significant tongue volume decrease in patients with controlled acromegaly (120+/−14 mL, *P* < .05) in comparison with the persistent uncontrolled group of patients (137+/−10 mL, *P* = not significant) after treatment with octreotide (268). In the same line, one prospective study with 30 patients with newly diagnosed acromegaly concluded that presurgical therapy with lanreotide autogel lowers GH and IGF-1 concentrations, induces tumor shrinkage, and ameliorates/reverses cardiac, vascular, and sleep complications in many patients with acromegaly (270). However, they found that unexpectedly, despite almost universal improvements in biochemical control (GH and IGF1 reductions in 93% of the cases), apnea hypopnea index responses to SRL therapy varied markedly among individuals: 14 (61%) exhibited an improvement and 2 (8.7%) showed no change, but 7 (30.4%) manifested a significant deterioration in obstructive SAS. Thus, although in general improvement of obstructive SAS is expected, it is difficult to identify which patients will improve. Nevertheless, the current guidelines support the use of presurgical therapy with SRLs for patients with severe pharyngeal thickness and sleep apnea, or high-output heart failure, with the aim of reducing surgical risk from severe comorbidities (1). There is no available information about the impact of pasireotide on SAS, but due to the mechanism of action, a similar or even higher effect than first generation SRL should be expected. Another study with 12 patients treated with pegvisomant reported that tongue volume decreased (105+/−33 to 83+/−33 mL; *P* = .007) as well as the apnea–hypopnea index (23+/−22 to 18+/−18/h; *P* = .0066) after treatment with pegvisomant (271).

## Management of cardiovascular and metabolic comorbidities in acromegaly

### General recommendations: lifestyle and non-pharmacological interventions

As there is no specific recommendation for patients with acromegaly, lifestyle, and non-pharmacological interventions in acromegaly patients should follow the general recommendations as for any patient with increased CV risk (142): sodium restriction (<2.3 g/day) and increased potassium intake, except in patients with chronic kidney disease; weight management is essential, particularly after successful acromegaly treatment, to prevent metabolic complications; aerobic and resistance exercise (≥150 minutes/week of moderate intensity) to improve endothelial function and reduce BP; management of obstructive SAS, which is highly prevalent in acromegaly and can contribute to BP elevation and smoking cessation. As recommended for patients with diabetes in general, lifestyle interventions are essential for proper control of acromegaly-related diabetes or glucose abnormalities. These positive health behaviors include diabetes self-management education and support, medical nutrition therapy, routine physical activity, adequate quality sleep, support for cessation of tobacco products, health behavior counseling, and psychosocial care (272).

### Management of hypertension

**Figure 6:**
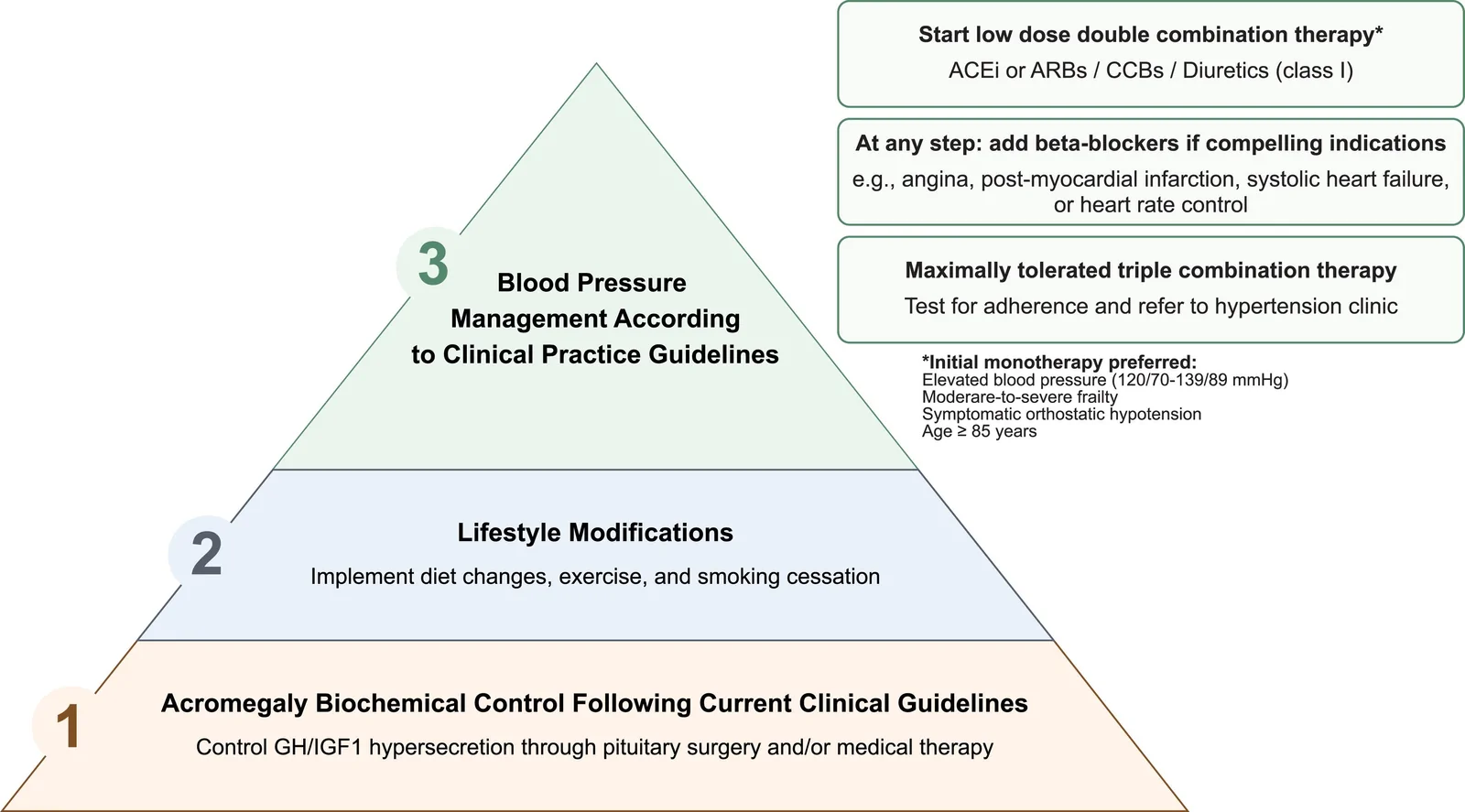
Treatment algorithm for hypertension in acromegaly. This flowchart outlines a stepwise approach to managing hypertension in acromegaly, integrating biochemical control, lifestyle modifications, and blood pressure (BP) management. The algorithm emphasizes the importance of growth hormone (GH) and insulin-like growth factor 1 (IGF-1) normalization through surgery or medical therapy, followed by lifestyle modification. Hypertension treatment follows current clinical guidelines, starting with low-dose combination therapy and escalating as needed. Beta-blockers are added for specific indications. ACEi, angiotensin-converting enzyme inhibitor; ARB, angiotensin II receptor blocker; BP, blood pressure; CCB, calcium channel blocker; GH, growth hormone; IGF-1, insulin-like growth factor 1. Recommendations are based on guidelines from the general population as there is no specific data in acromegaly (142).

There are no studies specifically addressing the medical treatment of hypertension in acromegaly. Thus, the selection of antihypertensive medications should follow similar principles as for the general population, with no specific antihypertensive drug class being universally recommended in acromegaly (Table S5 and Fig. 6) (58, 141).

The primary antihypertensive drug classes with strong evidence for reducing CV events through BP control in the general population include angiotensin-converting enzyme inhibitors (ACEi), angiotensin receptor blockers (ARBs), dihydropyridine calcium channel blockers (CCBs), thiazide or thiazide-like diuretics (such as hydrochlorothiazide, chlorthalidone, and indapamide), and β-blockers (142). ACEIs are a good option for those patients with LVH or proteinuria, and ARBs are an alternative to ACEIs with similar benefits. Moreover, they are effective in reducing LVH, a frequent complication in patients with acromegaly (7). The first four medications are normally recommended as first-line, while β-blockers are recommended in specific circumstances, such as angina, heart failure, post-myocardial infarction, or heart rate control. Additionally, β-blockers and diuretics may increase the risk of new-onset diabetes mellitus in predisposed patients, such as acromegaly patients (273). However, it is worth noting that some experts recommend diuretics (mainly ENaC-targeting agents) and β-blockers as a first choice in acromegaly, based on the pathophysiological mechanisms unique to this condition (42, 274). Another promising antihypertensive medication for patients with acromegaly are sodium-glucose co-transporter 2 (SGLT2) inhibitors, especially for those cases with diabetes and metabolic syndrome (275). However, SGLT2 inhibitors should be considered cautiously among partially controlled acromegaly patients and not recommended in poorly controlled patients due to the risk of diabetic ketoacidosis (276).

Real-world data from the recent Iberian acromegaly study showed that the most used drug classes were ACEIs (40.2%) and ARBs (37.3%), followed by thiazide diuretics (34.9%) and CCBs (22.5%), while β-blockers were used in 18.2% of the cases, mirroring the above-mentioned recommendations concerning first-line antihypertensive drugs (122).

In addition, as recommended for the general hypertensive population (142), combined antihypertensive therapy may be also advisable for acromegaly patients with hypertension, except in specific situations detailed in Fig. 6. This approach enhances treatment efficacy, likely due to the ability of targeting multiple physiological mechanisms underlying acromegaly-related hypertension, as well as it minimizes the occurrence of side effects as lower doses of individual antihypertensive drugs are used (142).

### Management of cardiovascular disease

In relation to cardiomyopathy, it is reported that it can be reversed if the diagnosis is made early in the course of the disease. The management of heart failure should follow standard guidelines for heart failure, and thus, ACEi, ARBs, β-blockers, and mineralocorticoid receptor antagonists are useful (9, 161). For arrhythmia management, it is important to ensure optimal control of heart rate and consider the use of devices such as implantable automatic defibrillators or pacemakers, especially for patients with advanced atrioventricular block. Moreover, catheter ablation should be considered in patients with atrial fibrillation to improve symptoms, quality of life, and ventricular function (39, 161).

Atherosclerosis may be better managed by targeting commonly recognized CV risk factors (smoking status, insulin resistance, hypertension, etc.) (9). The main therapeutic interventions focused on improving CV prognosis in acromegaly patients are described in Table S6 (58). However, it should be noted that those patients with clinically relevant heart failure, arrhythmia or CAD, should be referred to and treated by a dedicated cardiologist.

### Management of diabetes and dyslipidemia

The management of diabetes in acromegaly presents unique therapeutic challenges, requiring individualized approaches that consider the underlying pathophysiology of GH/IGF-1 excess (98, 99). First-line treatment should aim at achieving biochemical control of GH and IGF-1, as normalization of hormonal excess has been shown to improve insulin sensitivity and glucose metabolism (103). However, certain considerations are necessary when surgery fails to achieve GH and IGF-1 normalization. In such cases, the choice of pharmacologic therapy for acromegaly should take into account the potential metabolic impact, as some agents may negatively affect glucose homeostasis (250, 277). When antihyperglycemic therapy is required, the treatment approach generally follows standard diabetes guidelines for the general population (278). Metformin remains the preferred first-line option due to its effectiveness in lowering hepatic glucose production, improving insulin sensitivity, and a favorable cost-effectiveness profile (98, 99). Newer agents such as glucagon-like peptide-1 receptor agonists and SGLT2 inhibitors may provide similar cardiometabolic important benefits as in type 2 diabetes. However, the distinct metabolic characteristics of acromegaly, including increased ketogenesis and potential interactions between incretins and GH secretion, should be taken into account, as they may influence therapeutic responses and safety (99).

Regarding dyslipidemia, while acromegaly is associated with altered lipoprotein metabolism (12), current treatment recommendations for managing dyslipidemia are largely based on general cardiovascular risk management guidelines, as there are no acromegaly-specific lipid treatment protocols (209).

## Gaps in current knowledge and future directions

Despite advances in understanding the CV burden of acromegaly, several important gaps remain. Most current recommendations are extrapolated from the general population, without specifically accounting for the unique pathophysiological features of GH/IGF-1 excess and the typical acromegaly-related CV phenotype. Prospective, longitudinal studies with well-defined CV endpoints are still lacking. In particular, there is a need for standardized protocols for cardiovascular screening across disease stages, especially for subclinical manifestations and for a better characterization of atherosclerotic risk, given the controversy in relation to the prevalence of CAD. In addition, a proper evaluation of the real risk of arrhythmia and sudden cardiac death of patients with acromegaly is needed, as there is no clear evidence between the link of GH/IGF-1 excess and the risk of arrhythmia and sudden cardiac death. Prospective studies focused on elucidating the real prevalence of cardiac valve disease and its clinical repercussion on patients with acromegaly should be performed, and randomized trials assessing CV outcomes in response to acromegaly-specific treatments (surgery, fgSRLs, pegvisomant, pasireotide) will be useful to determine the real impact of these therapies on CV comorbidities. We also consider that the identification of predictive biomarkers for CV complications and treatment response would help to stratify the CV risk of our patients with acromegaly and help to determine the most suitable treatment in each patient.

The future trajectory of clinical management for hypertension and associated CV complications in acromegaly necessitates a rigorous, multi-pronged research strategy. Moving beyond current generalized endocrinological and CV recommendations requires dedicated studies focusing on specific pathophysiological mechanisms, therapeutic efficacy, and risk stratification unique to this disorder. Prospective cohort studies are foundational for understanding the natural history of hypertension development, progression, and response to systemic control in acromegaly, which is often poorly captured by retrospective reviews. In addition, the temporal relationship between biochemical normalization and CV risk reduction is often assumed but requires precise longitudinal quantification. In this regard, studies must explicitly link the duration and stability of controlled disease status to hard cardiovascular endpoints. Furthermore, the identification of molecular biomarkers capable of predicting imminent CV decompensation or identifying responders to specific therapies is paramount for personalized medicine. Research initiatives and efforts covering these aspects will be essential to move from general recommendations to acromegaly-specific clinical algorithms regarding the management of hypertension and CV disease in acromegaly, which ultimately will improve the CV risk management and clinical outcomes of acromegaly patients with hypertension and/or cardiomyopathy.

## Conclusions

CV disease remains a leading cause of morbidity and mortality in patients with acromegaly, necessitating a sophisticated understanding of the interplay between hormonal excess and vascular pathology. Although the biochemical control of GH and IGF-1 significantly improves many cardiac and vascular abnormalities, a substantial residual CV risk persists. This ongoing vulnerability is frequently driven by permanent structural changes, the cumulative impact of long-standing comorbidities, and the deleterious effects of diagnostic delays that allow cardiovascular remodeling to become irreversible. Consequently, early and systematic screening, individualized treatment of CV comorbidities, and the implementation of rigorous multidisciplinary care are critical to optimizing patient outcomes. Ongoing research must now transition toward a more granular exploration of disease-specific CV pathways, moving beyond general population guidelines to address the unique pathophysiology of the acromegalic heart. To achieve this, the scientific community must prioritize the initiation of large-scale prospective cohort studies designed to track the natural history of vascular complications under modern therapeutic regimens. These longitudinal observations are essential for evaluating the long-term impact of stringent GH/IGF-1 control on systemic blood pressure and hard CV endpoints, such as myocardial infarction, heart failure, and stroke.

Furthermore, there is an urgent clinical requirement for targeted intervention trials specifically tailored to evaluate the efficacy of various anti-hypertensive classes within this population. Such trials will clarify whether certain pharmacological agents offer superior cardioprotection in the context of GH excess, thereby moving away from empirical prescribing. Parallel to these clinical trials, research efforts should be directed toward the identification and validation of novel biomarkers and clinical predictors. The discovery of these indicators is paramount for sophisticated cardiovascular risk stratification, allowing clinicians to distinguish between patients at low risk and those requiring intensive, early intervention.

## Abbreviations

ABPM: ambulatory blood pressure monitoring
ACEi: angiotensin-converting enzyme inhibitors
ARB: angiotensin receptor blockers
BMI: body mass index
CAD: coronary artery disease
CCB: calcium channel blockers
CT: computed tomography
CV: cardiovascular
ECG: electrocardiogram
ENaC: epithelial sodium channel
GH: growth hormone
HBPM: home blood pressure monitoring
HDL: high-density lipoprotein
IGF-1: insulin-like growth factor-1
LDL: low-density lipoprotein
LVH: left ventricular hypertrophy
LVM: left ventricular mass
OR: odds ratio
PAI-1: plasminogen activator inhibitor-1
SAS: sleep apnea syndrome
SMR: standardized mortality risk

## References

[1] Katznelson L, Laws ER, Melmed S, et al Acromegaly: an endocrine society clinical practice guideline. J Clin Endocrinol Metab. 2014;99(11):3933‐3951. 25356808 10.1210/jc.2014-2700

[2] Melmed S, di Filippo L, Fleseriu M, et al Consensus on acromegaly therapeutic outcomes: an update. Nat Rev Endocrinol. 2025;21(11):718‐737. 40804505 10.1038/s41574-025-01148-2

[3] Crisafulli S, Luxi N, Sultana J, et al Global epidemiology of acromegaly: a systematic review and meta-analysis. Eur J Endocrinol. 2021;185(2):251‐263. 34061771 10.1530/EJE-21-0216

[4] Ohno H, Yoneoka Y, Jinguji S, Watanabe N, Okada M, Fujii Y. Has acromegaly been diagnosed earlier? J Clin Neurosci. 2018;48:138‐142. 29113856 10.1016/j.jocn.2017.10.086

[5] Lavrentaki A, Paluzzi A, Wass JAH, Karavitaki N. Epidemiology of acromegaly: review of population studies. Pituitary. 2017;20(1):4‐9. 27743174 10.1007/s11102-016-0754-x PMC5334410

[6] Wolf P, Maione L, Kamenický P, Chanson P. Cardiomyopathy in patients with acromegaly—not truly a concern anymore? J Clin Endocrinol Metab. 2025;110(10):2718‐2728. 40488310 10.1210/clinem/dgaf338

[7] Slagboom TNA, van Bunderen CC, De Vries R, Bisschop PH, Drent ML. Prevalence of clinical signs, symptoms and comorbidities at diagnosis of acromegaly: a systematic review in accordance with PRISMA guidelines. Pituitary. 2023;26(4):319‐332. 37210433 10.1007/s11102-023-01322-7 PMC10397145

[8] Colao A, Ferone D, Marzullo P, Lombardi G. Systemic complications of acromegaly: epidemiology, pathogenesis, and management. Endocr Rev. 2004;25(1):102‐152. 14769829 10.1210/er.2002-0022

[9] Iglesias P . Acromegaly and cardiovascular disease: associated cardiovascular risk factors, cardiovascular prognosis, and therapeutic impact. J Clin Med. 2025;14(6):1906. 40142714 10.3390/jcm14061906 PMC11943432

[10] Ramos-Leví AM, Marazuela M. Bringing cardiovascular comorbidities in acromegaly to an update. How should we diagnose and manage them? Front Endocrinol (Lausanne). 2019;10:120. 30930848 10.3389/fendo.2019.00120 PMC6423916

[11] Colao A, Pivonello R, Galderisi M, et al Impact of treating acromegaly first with surgery or somatostatin analogs on cardiomyopathy. J Clin Endocrinol Metab. 2008;93(7):2639‐2646. 18445662 10.1210/jc.2008-0299

[12] Briet C, Ilie MD, Kuhn E, et al Changes in metabolic parameters and cardiovascular risk factors after therapeutic control of acromegaly vary with the treatment modality. Data from the Bicêtre cohort, and review of the literature. Endocrine. 2019;63(2):348‐360. 30397873 10.1007/s12020-018-1797-8

[13] Vila G, Luger A, van der Lely AJ, et al Hypertension in acromegaly in relationship to biochemical control and mortality: global ACROSTUDY outcomes. Front Endocrinol (Lausanne). 2020;11:. 10.3389/fendo.2020.577173 PMC7734123 33329385

[14] Colao A, Cuocolo A, Marzullo P, et al Is the acromegalic cardiomyopathy reversible? Effect of 5-year normalization of growth hormone and insulin-like growth factor I levels on cardiac performance. J Clin Endocrinol Metab. 2001;86(4):1551‐1557. 11297582 10.1210/jcem.86.4.7376

[15] Minniti G, Moroni C, Jaffrain-Rea ML, et al Marked improvement in cardiovascular function after successful transsphenoidal surgery in acromegalic patients. Clin Endocrinol (Oxf). 2001;55(3):307‐313. 11589673 10.1046/j.1365-2265.2001.01343.x

[16] Maison P, Tropeano AI, Macquin-Mavier I, Giustina A, Chanson P. Impact of somatostatin analogs on the heart in acromegaly: a metaanalysis. J Clin Endocrinol Metab. 2007;92(5):1743‐1747. 17311857 10.1210/jc.2006-2547

[17] Pivonello R, Galderisi M, Auriemma RS, et al Treatment with growth hormone receptor antagonist in acromegaly: effect on cardiac structure and performance. J Clin Endocrinol Metab. 2007;92(2):476‐482. 17105844 10.1210/jc.2006-1587

[18] Marie P . Sur deux cas d’acromégalie: hypertrophie singulière non congénitale des extrémités supérieures, inférieures et céphalique. ScienceOpen. Rev Med Liege. 1886. Accessed July 29, 2025. https://www.scienceopen.com/document?vid=3db2e398-9b05-43dc-8b3a-3ae786c3c32f

[19] Lie JT, Grossman SJ. Pathology of the heart in acromegaly: anatomic findings in 27 autopsied patients. Am Heart J. 1980;100(1):41‐52. 6446234 10.1016/0002-8703(80)90277-x

[20] Rodrigues EA, Caruana MP, Lahiri A, Nabarro JDN, Jacobs HS, Raftery EB. Subclinical cardiac dysfunction in acromegaly: evidence for a specific disease of heart muscle. Br Heart J. 1989;62(3):185‐194. 2528980 10.1136/hrt.62.3.185 PMC1216761

[21] Wright AD, Hill DM, Lowy C, Fraser TR. Mortality in acromegaly. Q J Med. 1970;39(153):1‐16. 5427331

[22] Dekkers OM, Biermasz NR, Pereira AM, Romijn JA, Vandenbroucke JP. Mortality in acromegaly: a metaanalysis. J Clin Endocrinol Metab. 2008;93(1):61‐67. 17971431 10.1210/jc.2007-1191

[23] Dal J, Feldt-Rasmussen U, Andersen M, et al Acromegaly incidence, prevalence, complications and long-term prognosis: a nationwide cohort study. Eur J Endocrinol. 2016;175(3):181‐190. 27280374 10.1530/EJE-16-0117

[24] Ritvonen E, Löyttyniemi E, Jaatinen P, et al Mortality in acromegaly: a 20-year follow-up study. Endocr Relat Cancer. 2016;23(6):469‐480. 27185871 10.1530/ERC-16-0106

[25] Esposito D, Ragnarsson O, Granfeldt D, Marlow T, Johannsson G, Olsson DS. Decreasing mortality and changes in treatment patterns in patients with acromegaly from a nationwide study. Eur J Endocrinol. 2018;178(5):459‐469. 29483205 10.1530/EJE-18-0015

[26] Bolfi F, Neves AF, Boguszewski CL, Nunes-Nogueira VS. Mortality in acromegaly decreased in the last decade: a systematic review and meta-analysis. Eur J Endocrinol. 2018;179(1):59‐71. 29764907 10.1530/EJE-18-0255

[27] Orme S, McNally R, James PW, et al Increased mortality in acromegaly is due to vascular and respiratory disease and is normalised by control of GH levels-A retrospective analysis from the UK acromegaly register 1970-2016. Clin Endocrinol (Oxf). 2024;100(6):558‐564. 38652736 10.1111/cen.15060

[28] Alexander L, Appleton D, Hall R, Ross WM, Wilkinson R. Epidemiology of acromegaly in the Newcastle region. Clin Endocrinol (Oxf). 1980;12(1):71‐79. 7379316 10.1111/j.1365-2265.1980.tb03135.x

[29] Maione L, Chanson P. National acromegaly registries. Best Pract Res Clin Endocrinol Metab. 2019;33(2):101264. 30894298 10.1016/j.beem.2019.02.001

[30] Biagetti B, Iglesias P, Villar-Taibo R, et al Mortality in acromegaly diagnosed in older individuals in Spain is higher in women compared to the general Spanish population. J Clin Endocrinol Metab. 2023;108(9):2193‐2202. 36916151 10.1210/clinem/dgad141

[31] Mercado M, Gonzalez B, Vargas G, et al Successful mortality reduction and control of comorbidities in patients with acromegaly followed at a highly specialized multidisciplinary clinic. J Clin Endocrinol Metab. 2014;99(12):4438‐4446. 25210882 10.1210/jc.2014-2670

[32] Kauppinen-Mäkelin R, Sane T, Reunanen A, et al A nationwide survey of mortality in acromegaly. J Clin Endocrinol Metab. 2005;90(7):4081‐4086. 15886256 10.1210/jc.2004-1381

[33] Esposito D, Ragnarsson O, Johannsson G, Olsson DS. Prolonged diagnostic delay in acromegaly is associated with increased morbidity and mortality. Eur J Endocrinol. 2020;182(6):523‐531. 32213651 10.1530/EJE-20-0019

[34] Rosendal C, Arlien-Søborg MC, Nielsen EH, et al The changing landscape of acromegaly—an epidemiological perspective. Rev Endocr Metab Disord. 2024;25(4):691‐705. 38337125 10.1007/s11154-024-09875-z

[35] Hoskuldsdottir GT, Fjalldal SB, Sigurjonsdottir HA. The incidence and prevalence of acromegaly, a nationwide study from 1955 through 2013. Pituitary. 2015;18(6):803‐807. 25893613 10.1007/s11102-015-0655-4

[36] Caputo M, Ucciero A, Mele C, et al Use of administrative health databases to estimate incidence and prevalence of acromegaly in piedmont region, Italy. J Endocrinol Invest. 2019;42(4):397‐402. 30069856 10.1007/s40618-018-0928-7

[37] Fauchier G, Laurent E, Maione L, et al Acromegaly: incidence, patient characteristics and treatment patterns in a 10-year nationwide retrospective hospital cohort study. Ann Endocrinol (Paris). 2024;85(6):589‐595. 39307237 10.1016/j.ando.2024.09.002

[38] Jallad RS, Bronstein MD. Acromegaly in the elderly patient. Arch Endocrinol Metab. 2019;63(6):638‐645. 31939489 10.20945/2359-3997000000194 PMC10522238

[39] Mosca S, Paolillo S, Colao A, et al Cardiovascular involvement in patients affected by acromegaly: an appraisal. Int J Cardiol. 2013;167(5):1712‐1718. 23219077 10.1016/j.ijcard.2012.11.109

[40] Isgaard J, Arcopinto M, Karason K, Cittadini A. GH and the cardiovascular system: an update on a topic at heart. Endocrine. 2015;48(1):25‐35. 24972804 10.1007/s12020-014-0327-6 PMC4328125

[41] Colao A, Grasso LFS, Di Somma C, Pivonello R. Acromegaly and heart failure. Heart Fail Clin. 2019;15(3):399‐408. 31079698 10.1016/j.hfc.2019.03.001

[42] Kamenicky P, Blanchard A, Frank M, et al Body fluid expansion in acromegaly is related to enhanced epithelial sodium channel (ENaC) activity. J Clin Endocrinol Metab. 2011;96(7):2127‐2135. 21508131 10.1210/jc.2011-0078

[43] Mulatero P, Veglio F, Maffei P, et al CYP11B2 -344T/C gene polymorphism and blood pressure in patients with acromegaly. J Clin Endocrinol Metab. 2006;91(12):5008‐5012. 17003099 10.1210/jc.2006-0049

[44] Bielohuby M, Roemmler J, Manolopoulou J, et al Chronic growth hormone excess is associated with increased aldosterone: a study in patients with acromegaly and in growth hormone transgenic mice. Exp Biol Med (Maywood). 2009;234(8):1002‐1009. 19491373 10.3181/0901-RM-34

[45] Bondanelli M, Ambrosio MR, degli Uberti EC. Pathogenesis and prevalence of hypertension in acromegaly. Pituitary. 2001;4(4):239‐249. 12501974 10.1023/a:1020798430884

[46] Lombardi G, Di Somma C, Grasso LFS, Savanelli MC, Colao A, Pivonello R. The cardiovascular system in growth hormone excess and growth hormone deficiency. J Endocrinol Invest. 2012;35(11):1021‐1029. 23143695 10.3275/8717

[47] Bohlooly-Y M, Carlson L, Olsson B, et al Vascular function and blood pressure in GH transgenic mice. Endocrinology. 2001;142(8):3317‐3323. 11459773 10.1210/endo.142.8.8296

[48] Gadelha MR, Kasuki L, Lim DSTT, Fleseriu M. Systemic complications of acromegaly and the impact of the current treatment landscape: an update. Endocr Rev. 2019;40(1):268‐332. 30184064 10.1210/er.2018-00115

[49] Clayton RN . Cardiovascular function in acromegaly. Endocr Rev. 2003;24(3):272‐277. 12788799 10.1210/er.2003-0009

[50] Wolf P, Maione L, Kamenický P, Chanson P. Acromegalic cardiomyopathy: an entity on its own? The effects of GH and IGF-I excess and treatment on cardiovascular risk factors. Arch Med Res. 2023;54(8):102921. 38040526 10.1016/j.arcmed.2023.102921

[51] Frustaci A, Chimenti C, Setoguchi M, et al Cell death in acromegalic cardiomyopathy. Circulation. 1999;99(11):1426‐1434. 10086965 10.1161/01.cir.99.11.1426

[52] Wolf P, Bouazizi K, Kachenoura N, et al Increase in intracellular and extracellular myocardial mass in patients with acromegaly: a cardiac magnetic resonance imaging study. Eur J Endocrinol. 2023;189(2):199‐207. 37549351 10.1093/ejendo/lvad105

[53] Gouya H, Vignaux O, Le Roux P, et al Rapidly reversible myocardial edema in patients with acromegaly: assessment with ultrafast T2 mapping in a single-breath-hold MRI sequence. Am J Roentgenol. 2008;190(6):1576‐1582. 18492909 10.2214/AJR.07.2031

[54] Winhofer Y, Wolf P, Krššák M, et al No evidence of ectopic lipid accumulation in the pathophysiology of the acromegalic cardiomyopathy. J Clin Endocrinol Metab. 2014;99(11):4299‐4306. 25148232 10.1210/jc.2014-2242

[55] Young JA, Zhu S, List EO, Duran-Ortiz S, Slama Y, Berryman DE. Musculoskeletal effects of altered GH action. Front Physiol. 2022;13:867921. 35665221 10.3389/fphys.2022.867921 PMC9160929

[56] Loher H, Jenni S, Bucher J, et al Impaired repletion of intramyocellular lipids in patients with growth hormone deficiency after a bout of aerobic exercise. Growth Horm IGF Res. 2018;42-43:32‐39. 30153529 10.1016/j.ghir.2018.08.001

[57] Ramos-Leví AM, Marazuela M. Cardiovascular comorbidities in acromegaly: an update on their diagnosis and management. Endocrine. 2017;55(2):346‐359. 28042644 10.1007/s12020-016-1191-3

[58] Araujo-Castro M . n.d. Supplementary material of cardiovascular disease and acromegaly. Zenodo. 10.5281/ZENODO.18051835

[59] Minniti G, Jaffrain-Rea ML, Moroni C, et al Echocardiographic evidence for a direct effect of GH/IGF-I hypersecretion on cardiac mass and function in young acromegalics. Clin Endocrinol (Oxf). 1998;49(1):101‐106. 9797853 10.1046/j.1365-2265.1998.00493.x

[60] Fazio S, Cittadini A, Biondi B, et al Cardiovascular effects of short-term growth hormone hypersecretion. J Clin Endocrinol Metab. 2000;85(1):179‐182. 10634384 10.1210/jcem.85.1.6313

[61] Bogazzi F, Lombardi M, Strata E, et al High prevalence of cardiac hypertophy without detectable signs of fibrosis in patients with untreated active acromegaly: an in vivo study using magnetic resonance imaging. Clin Endocrinol (Oxf). 2008;68(3):361‐368. 17854389 10.1111/j.1365-2265.2007.03047.x

[62] Dos Santos Silva CM, Gottlieb I, Volschan I, et al Low frequency of cardiomyopathy using cardiac magnetic resonance imaging in an acromegaly contemporary cohort. J Clin Endocrinol Metab. 2015;100(12):4447‐4455. 26431508 10.1210/jc.2015-2675

[63] Katholi RE, Couri DM. Left ventricular hypertrophy: major risk factor in patients with hypertension: update and practical clinical applications. Int J Hypertens. 2011;2011:1‐10. 10.4061/2011/495349 PMC3132610 21755036

[64] Colao A, Baldelli R, Marzullo P, et al Systemic hypertension and impaired glucose tolerance are independently correlated to the severity of the acromegalic cardiomyopathy 1. J Clin Endocrinol Metab. 2000;85(1):193‐199. 10634386 10.1210/jcem.85.1.6318

[65] Colao A, Spinelli L, Marzullo P, et al High prevalence of cardiac valve disease in acromegaly: an observational, analytical, case-control study. J Clin Endocrinol Metab. 2003;88(7):3196‐3201. 12843165 10.1210/jc.2002-021099

[66] Hinojosa-Amaya JM, Varlamov EV, Yedinak CG, et al Echocardiographic findings in acromegaly: prevalence of concentric left ventricular remodeling in a large single-center cohort. J Endocrinol Invest. 2021;44(12):2665‐2674. 33893617 10.1007/s40618-021-01579-4

[67] Pereira AM, Van Thiel SW, Lindner JR, et al Increased prevalence of regurgitant valvular heart disease in acromegaly. J Clin Endocrinol Metab. 2004;89(1):71‐75. 14715829 10.1210/jc.2003-030849

[68] Ságová I, Dragula M, Mokáň M, Vaňuga P. Filling the gap between the heart and the body in acromegaly: a case-control study. Endocrine. 2023;79(2):365‐375. 36309947 10.1007/s12020-022-03232-3 PMC9892104

[69] Nemes A, Kormányos Á, Ambrus N, Lengyel C, Valkusz Z. Myocardial, valvular, and vascular structural and functional properties in acromegaly. J Clin Med. 2023;12(21):6857. 37959322 10.3390/jcm12216857 PMC10648583

[70] Pivonello R, Auriemma RS, Grasso LFS, et al Complications of acromegaly: cardiovascular, respiratory and metabolic comorbidities. Pituitary. 2017;20(1):46‐62. 28224405 10.1007/s11102-017-0797-7

[71] Rabkin E, Aikawa M, Stone JR, Fukumoto Y, Libby P, Schoen FJ. Activated interstitial myofibroblasts express catabolic enzymes and mediate matrix remodeling in myxomatous heart valves. Circulation. 2001;104(21):2525‐2532. 11714645 10.1161/hc4601.099489

[72] Casini AF, Neto LV, Fontes R, França RF, Xavier SS, Gadelha MR. Aortic root ectasia in patients with acromegaly: experience at a single center. Clin Endocrinol (Oxf). 2011;75(4):495‐500. 21521335 10.1111/j.1365-2265.2011.04067.x

[73] Colao A, Grasso LFS. Aortic root ectasia in patients with acromegaly: an emerging complication. Clin Endocrinol (Oxf). 2011;75(4):420‐421. 21623859 10.1111/j.1365-2265.2011.04131.x

[74] Kahaly G, Olshausen K V, Mohr-Kahaly S, et al Arrhythmia profile in acromegaly. Eur Heart J. 1992;13(1):51‐56. 1577031 10.1093/oxfordjournals.eurheartj.a060047

[75] Kinugawa S, Tsutsui H, Ide T, et al Positive inotropic effect of insulin-like growth factor-1 on normal and failing cardiac myocytes. Cardiovasc Res. 1999;43(1):157‐164. 10536700 10.1016/s0008-6363(99)00058-9

[76] De Alcubierre D, Feola T, Cozzolino A, et al The spectrum of cardiac abnormalities in patients with acromegaly: results from a case-control cardiac magnetic resonance study. Pituitary. 2024;27(4):416‐427. 38847918 10.1007/s11102-024-01403-1 PMC11289141

[77] Landin-Wilhelmsen K, Tengborn L, Wilhelmsen L, Bengtsson BÅ. Elevated fibrinogen levels decrease following treatment of acromegaly. Clin Endocrinol (Oxf). 1997;46(1):69‐74. 9059560 10.1046/j.1365-2265.1997.d01-1743.x

[78] Frara S, Maffezzoni F, Mazziotti G, Giustina A. Current and emerging aspects of diabetes mellitus in acromegaly. Trends Endocrinol Metab. 2016;27(7):470‐483. 27229934 10.1016/j.tem.2016.04.014

[79] Maffei P, Dassie F, Wennberg A, Parolin M, Vettor R. The endothelium in acromegaly. Front Endocrinol (Lausanne). 2019;10:437. 31396153 10.3389/fendo.2019.00437 PMC6667653

[80] Ferns GAA, Motani AS, Anggard EE. The insulin-like growth factors: their putative role in atherogenesis. Artery. 1991;18(4):197‐225. 1714710

[81] Schöfl C, Petroff D, Tönjes A, et al Incidence of myocardial infarction and stroke in acromegaly patients: results from the German acromegaly registry. Pituitary. 2017;20(6):635‐642. 28808855 10.1007/s11102-017-0827-5

[82] Bogazzi F, Battolla L, Spinelli C, et al Risk factors for development of coronary heart disease in patients with acromegaly: a five-year prospective study. J Clin Endocrinol Metab. 2007;92(11):4271‐4277. 17785352 10.1210/jc.2007-1213

[83] Herrmann BL, Severing M, Schmermund A, et al Impact of disease duration on coronary calcification in patients with acromegaly. Exp Clin Endocrinol Diabetes. 2009;117(8):417‐422. 19373755 10.1055/s-0029-1214386

[84] Delafontaine P, Bernstein KE, Alexander RW. Insulin-like growth factor I gene expression in vascular cells. Hypertension. 1991;17(5):693‐699. 1708744 10.1161/01.hyp.17.5.693

[85] Limongelli G, Fioretti V, Di Maio M, et al Left atrial volume during stress is associated with increased risk of arrhythmias in patients with hypertrophic cardiomyopathy. J Cardiovasc Echogr. 2019;29(1):1‐6. 31008030 10.4103/jcecho.jcecho_45_18 PMC6450231

[86] Bar RS, Harrison LC, Baxter RC, et al Production of IGF-binding proteins by vascular endothelial cells. Biochem Biophys Res Commun. 1987;148(2):734‐739. 2961328 10.1016/0006-291x(87)90937-5

[87] Cercek B, Fishbein MC, Forrester JS, Helfant RH, Fagin JA. Induction of insulin-like growth factor I messenger RNA in rat aorta after balloon denudation. Circ Res. 1990;66(6):1755‐1760. 2188750 10.1161/01.res.66.6.1755

[88] Thum T, Tsikas D, Frölich JC, Borlak J. Growth hormone induces eNOS expression and nitric oxide release in a cultured human endothelial cell line. FEBS Lett. 2003;555(3):567‐571. 14675775 10.1016/s0014-5793(03)01356-5

[89] Erem C, Nuhoglu I, Kocak M, et al Blood coagulation and fibrinolysis in patients with acromegaly: increased plasminogen activator inhibitor-1 (PAI-1), decreased tissue factor pathway inhibitor (TFPI), and an inverse correlation between growth hormone and TFPI. Endocrine. 2008;33(3):270‐276. 19016004 10.1007/s12020-008-9088-4

[90] Manara R, Maffei P, Citton V, et al Increased rate of intracranial saccular aneurysms in acromegaly: an MR angiography study and review of the literature. J Clin Endocrinol Metab. 2011;96(5):1292‐1300. 21307138 10.1210/jc.2010-2721

[91] Kyriakakis N, Pechlivani N, Lynch J, et al Prothrombotic fibrin network characteristics in patients with acromegaly: a novel mechanism for vascular complications. Eur J Endocrinol. 2020;182(5):511‐521. 32197236 10.1530/EJE-19-0817

[92] Campello E, Marobin M, Barbot M, et al The haemostatic system in acromegaly: a single-centre case–control study. J Endocrinol Invest. 2020;43(7):1009‐1018. 31994012 10.1007/s40618-020-01186-9

[93] Kyriakakis N, Lynch J, Seejore K, et al SUN-075 higher fibrinogen and clot density in patients with acromegaly: the role of adverse body composition to the increased thrombotic potential. J Endocr Soc. 2019;3(Suppl 1):SUN-075.

[94] Vilar L, Naves LA, Costa SS, Abdalla LF, Coelho CE, Casulari LA. Increase of classic and nonclassic cardiovascular risk factors in patients with acromegaly. Endocr Pract. 2007;13(4):363‐372. 17669712 10.4158/EP.13.4.363

[95] Colak A, Yllmaz H, Temel Y, et al Coagulation parameters and platelet function analysis in patients with acromegaly. J Endocrinol Invest. 2016;39(1):97‐101. 26048595 10.1007/s40618-015-0300-0

[96] Sartorio A, Cattaneo M, Bucciarelli P, et al Alterations of haemostatic and fibrinolytic markers in adult patients with growth hormone deficiency and with acromegaly. Exp Clin Endocrinol Diabetes. 2000;108(7):486‐492. 11083070 10.1055/s-2000-8145

[97] Amado A, Araújo F, Carvalho D. Cardiovascular risk factors in acromegaly: what's the impact of disease control? Exp Clin Endocrinol Diabetes. 2018;126(8):505‐512. 29365332 10.1055/s-0043-124668

[98] Esposito D, Boguszewski CL, Colao A, et al Diabetes mellitus in patients with acromegaly: pathophysiology, clinical challenges and management. Nat Rev Endocrinol. 2024;20(9):541‐552. 38844688 10.1038/s41574-024-00993-x

[99] Biagetti B, Araujo-Castro M, Marazuela M, Puig-Domingo M. Treatment of acromegaly-induced diabetes: an updated proposal. Pituitary. 2025;28(1):15. 10.1007/s11102-024-01477-x 39738706

[100] Moustaki M, Paschou SA, Xekouki P, et al Secondary diabetes mellitus in acromegaly. Endocrine. 2023;81(1):1‐15. 36882643 10.1007/s12020-023-03339-1 PMC10239382

[101] Biagetti B, Simó R. GH/IGF-1 abnormalities and muscle impairment: from basic research to clinical practice. Int J Mol Sci. 2021;22(1):415. 33401779 10.3390/ijms22010415 PMC7795003

[102] Ferraù F, Albani A, Ciresi A, Giordano C, Cannavò S. Diabetes secondary to acromegaly: physiopathology, clinical features and effects of treatment. Front Endocrinol (Lausanne). 2018;9:358. 30034367 10.3389/fendo.2018.00358 PMC6043782

[103] Pascual-Corrales E, Biagetti B, Marazuela M, et al Glucose metabolism outcomes after pituitary surgery in patients with acromegaly. Pituitary. 2024;27(5):497‐506. 38940859 10.1007/s11102-024-01415-x

[104] Wolters TLC, Netea MG, Riksen NP, Hermus ARMM, Netea-Maier RT. Acromegaly, inflammation and cardiovascular disease: a review. Rev Endocr Metab Disord. 2020;21(4):547‐568. 32458292 10.1007/s11154-020-09560-x PMC7560935

[105] Giordano C, Ciresi A, Amato MC, et al Clinical and metabolic effects of first-line treatment with somatostatin analogues or surgery in acromegaly: a retrospective and comparative study. Pituitary. 2012;15(4):539‐551. 22116639 10.1007/s11102-011-0365-5

[106] Ciresi A, Amato MC, Pivonello R, et al The metabolic profile in active acromegaly is gender-specific. J Clin Endocrinol Metab. 2013;98(1):E51‐E59. 23162101 10.1210/jc.2012-2896

[107] Wildbrett J, Hanefeld M, Fücker K, et al Anomalies of lipoprotein pattern and fibrinolysis in acromegalic patients: relation to growth hormone levels and insulin-like growth factor I. Exp Clin Endocrinol Diabetes. 1997;105(6):331‐335. 9439928 10.1055/s-0029-1211774

[108] Puglisi S, Ferraù F, Ragonese M, Spagnolo F, Cannavò S. Cardiometabolic risk in acromegaly: a review with a focus on pasireotide. Front Endocrinol (Lausanne). 2020;11:28. 32117056 10.3389/fendo.2020.00028 PMC7017075

[109] Dal J, List EO, Jørgensen JOL, Berryman DE. Glucose and fat metabolism in acromegaly: from mice models to patient care. Neuroendocrinology. 2016;103(1):96‐105. 25925240 10.1159/000430819

[110] Guo X, Gao L, Zhao Y, et al Characteristics of the upper respiratory tract in patients with acromegaly and correlations with obstructive sleep apnoea/hypopnea syndrome. Sleep Med. 2018;48:27‐34. 29852361 10.1016/j.sleep.2018.04.011

[111] Rodrigues MP, Naves LA, Casulari LA, et al Craniofacial abnormalities, obesity, and hormonal alterations have similar effects in magnitude on the development of nocturnal hypoxemia in patients with acromegaly. J Endocrinol Invest. 2008;31(12):1052‐1057. 19246969 10.1007/BF03345651

[112] Evans CC, Hipkin LJ, Murray GM. Pulmonary function in acromegaly. Thorax. 1977;32(3):322‐327. 882947 10.1136/thx.32.3.322 PMC470609

[113] Trotman-Dickenson B, Weetman A, Hughes J. Upper airflow obstruction and pulmonary function in acromegaly: relationship to disease activity. Q J Med. 1991;79:527‐538. 1946933

[114] Luboshitzky R, Barzilai D. Hypoxemia and pulmonary function in acromegaly. Am Rev Respir Dis. 1980;121(3):471‐475. 6774634 10.1164/arrd.1980.121.3.471

[115] Weiss V, Šonka K, Pretl M, et al Prevalence of the sleep apnea syndrome in acromegaly population. J Endocrinol Invest. 2000;23(8):515‐519. 11021767 10.1007/BF03343767

[116] Dostálová S, Šonka K, Šmahel Z, Weiss V, Marek J, Hořínek D. Craniofacial abnormalities and their relevance for sleep apnoea syndrome aetiopathogenesis in acromegaly. Eur J Endocrinol. 2001;144:491‐497. 11331215 10.1530/eje.0.1440491

[117] Castellani C, Francia G, Dalle Carbonare L, et al Morphological study of upper airways and long-term follow-up of obstructive sleep apnea syndrome in acromegalic patients. Endocrine. 2016;51(2):308‐316. 26093846 10.1007/s12020-015-0659-x

[118] Davi MV, Dalle Carbonare L, Giustina A, et al Sleep apnoea syndrome is highly prevalent in acromegaly and only partially reversible after biochemical control of the disease. Eur J Endocrinol. 2008;159(5):533‐540. 18765561 10.1530/EJE-08-0442

[119] Vannucci L, Luciani P, Gagliardi E, et al Assessment of sleep apnea syndrome in treated acromegalic patients and correlation of its severity with clinical and laboratory parameters. J Endocrinol Invest. 2013;36(4):237‐242. 22776855 10.3275/8513

[120] Attal P, Chanson P. Endocrine aspects of obstructive sleep apnea. J Clin Endocrinol Metab. 2010;95(2):483‐495. 20061419 10.1210/jc.2009-1912

[121] Araujo-Castro M, Biagetti B, Menéndez Torre E, et al Differences between GH- and PRL-cosecreting and GH-secreting pituitary adenomas: a series of 604 cases. J Clin Endocrinol Metab. 2024;109(12):e2178‐e2187. 38436926 10.1210/clinem/dgae126

[122] Araujo-Castro M, García-Centeno R, González L, et al Prevalence and evolution of hypertension in a large Iberian cohort of patients with acromegaly. Pituitary. 2025;28(6):119. 41139153 10.1007/s11102-025-01586-1

[123] Arosio M, Reimondo G, Malchiodi E, et al Predictors of morbidity and mortality in acromegaly: an Italian survey. Eur J Endocrinol. 2012;167(2):189‐198. 22596288 10.1530/EJE-12-0084

[124] Vallette S, Ezzat S, Chik C, et al Emerging trends in the diagnosis and treatment of acromegaly in Canada. Clin Endocrinol (Oxf). 2013;79(1):79‐85. 23190441 10.1111/cen.12112

[125] Schöfl C, Franz H, Grussendorf M, et al Long-term outcome in patients with acromegaly: analysis of 1344 patients from the German acromegaly register. Eur J Endocrinol. 2013;168(1):39‐47. 23087126 10.1530/EJE-12-0602

[126] Kwon O, Song YD, Kim SY, Lee EJ. Nationwide survey of acromegaly in South Korea. Clin Endocrinol (Oxf). 2013;78(4):577‐585. 22909047 10.1111/cen.12020

[127] Howlett TA, Willis D, Walker G, Wass JAH, Trainer PJ. Control of growth hormone and IGF1 in patients with acromegaly in the UK: responses to medical treatment with somatostatin analogues and dopamine agonists. Clin Endocrinol (Oxf). 2013;79(5):689‐699. 23574573 10.1111/cen.12207

[128] Portocarrero-Ortiz LA, Vergara-Lopez A, Vidrio-Velazquez M, et al The Mexican acromegaly registry: clinical and biochemical characteristics at diagnosis and therapeutic outcomes. J Clin Endocrinol Metab. 2016;101(11):3997‐4004. 27428551 10.1210/jc.2016-1937

[129] Vila G, Dobnig H, Knosp E, et al Gender aspects in the biochemical control of acromegaly in Austria: evaluation of 607 cases from the Austrian acromegaly registry. Endocr Abstr. 2016;41.

[130] Maione L, Brue T, Beckers A, et al Changes in the management and comorbidities of acromegaly over three decades: the French acromegaly registry. Eur J Endocrinol. 2017;176(5):645‐655. 28246150 10.1530/EJE-16-1064

[131] Costenaro F, Martin A, Horn RF, Czepielewski MA, Rodrigues TC. Role of ambulatory blood pressure monitoring in patients with acromegaly. J Hypertens. 2016;34(7):1357‐1363. 27153464 10.1097/HJH.0000000000000962

[132] Minniti G, Moroni C, Jaffrain-Rea ML, et al Prevalence of hypertension in acromegalic patients: clinical measurement versus 24-hour ambulatory blood pressure monitoring. Clin Endocrinol (Oxf). 1998;48(2):149‐152. 9579225

[133] Vitale G, Pivonello R, Auriemma RS, et al Hypertension in acromegaly and in the normal population: prevalence and determinants. Clin Endocrinol (Oxf). 2005;63(4):470‐476. 16181242 10.1111/j.1365-2265.2005.02370.x

[134] Terzolo M, Matrella C, Boccuzzi A, et al Twenty-four hour profile of blood pressure in patients with acromegaly. Correlation with demographic, clinical and hormonal features. J Endocrinol Invest. 1999;22(1):48‐54. 10.1007/BF03345478 10090137

[135] Sardella C, Urbani C, Lombardi M, et al The beneficial effect of acromegaly control on blood pressure values in normotensive patients. Clin Endocrinol (Oxf). 2014;81(4):573‐581. 24661019 10.1111/cen.12455

[136] Arici FN, Gulumsek E, Ozturk HA, Avci BS, Sumbul HE. The relationship between morning blood pressure surge and serum IGF-1 level in acromegaly patients with hypertension. Ir J Med Sci. 2024;193(1):233‐240. 37498476 10.1007/s11845-023-03478-4

[137] Melmed S . Acromegaly and cancer: not a problem? J Clin Endocrinol Metab. 2001;86(7):2929‐2934. 11443145 10.1210/jcem.86.7.7635

[138] Sherlock M, Ayuk J, Tomlinson JW, et al Mortality in patients with pituitary disease. Endocr Rev. 2010;31(3):301‐342. 20086217 10.1210/er.2009-0033

[139] Rajasoorya C, Holdaway IM, Wrightson P, Scott DJ, Ibbertson HK. Determinants of clinical outcome and survival in acromegaly. Clin Endocrinol (Oxf). 1994;41(1):95‐102. 8050136 10.1111/j.1365-2265.1994.tb03789.x

[140] Holdaway IM, Rajasoorya CR, Gamble GD, Stewart AW. Long-term treatment outcome in acromegaly. Growth Horm IGF Res. 2003;13(4):185‐192. 12914751 10.1016/s1096-6374(03)00030-3

[141] Giustina A, Barkan A, Beckers A, et al A consensus on the diagnosis and treatment of acromegaly comorbidities: an update. J Clin Endocrinol Metab. 2020;105(4):E937‐E946. 10.1210/clinem/dgz096 31606735

[142] McCarthy CP, Bruno RM, Brouwers S, et al 2024 ESC Guidelines for the management of elevated blood pressure and hypertension: developed by the task force on the management of elevated blood pressure and hypertension of the European Society of Cardiology (ESC) and endorsed by the European Society of Endocrinology (ESE) and the European Stroke Organisation (ESO). Eur Heart J. 2024;45(38):3912‐4018. 39210715 10.1093/eurheartj/ehae178

[143] Guirguis-Blake JM, Evans C V, Webber EM, Coppola EL, Perdue LA, Weyrich MS. Screening for hypertension in adults: updated evidence report and systematic review for the US preventive services task force. JAMA. 2021;325(16):1657‐1669. 33904862 10.1001/jama.2020.21669 PMC13344483

[144] McEvoy JW, Leahy N, Parati G. The apples and oranges of blood pressure variability. Hypertension. 2023;80(12):2556‐2558. 37967158 10.1161/HYPERTENSIONAHA.123.21927

[145] Rocha P, Barroso J, Carlos F, Muxfeldt E, Gadelha M, Kasuki L. Importance of 24 h ambulatory blood pressure monitoring in patients with acromegaly and correlation with cardiac magnetic resonance findings. Pituitary. 2023;26(4):402‐410. 37247075 10.1007/s11102-023-01321-8

[146] Palmeiro CR, Anand R, Dardi IK, Balasubramaniyam N, Schwarcz MD, Weiss IA. Growth hormone and the cardiovascular system. Cardiol Rev. 2012;20(4):197‐207. 22314142 10.1097/CRD.0b013e318248a3e1

[147] Hong S, Kim KS, Han K, Park CY. Acromegaly and cardiovascular outcomes: a cohort study. Eur Heart J. 2022;43(15):1491‐1499. 34864952 10.1093/eurheartj/ehab822

[148] Colao A . The GH-IGF-I axis and the cardiovascular system: clinical implications. Clin Endocrinol (Oxf). 2008;69(3):347‐358. 18462260 10.1111/j.1365-2265.2008.03292.x

[149] Grothues F, Smith GC, Moon JCC, et al Comparison of interstudy reproducibility of cardiovascular magnetic resonance with two-dimensional echocardiography in normal subjects and in patients with heart failure or left ventricular hypertrophy. Am J Cardiol. 2002;90(1):29‐34. 12088775 10.1016/s0002-9149(02)02381-0

[150] Moon JCC, McKenna WJ, McCrohon JA, Elliott PM, Smith GC, Pennell DJ. Toward clinical risk assessment in hypertrophic cardiomyopathy with gadolinium cardiovascular magnetic resonance. J Am Coll Cardiol. 2003;41(9):1561‐1567. 12742298 10.1016/s0735-1097(03)00189-x

[151] Bernabeu I, Aller J, Álvarez-Escolá C, et al Criteria for diagnosis and postoperative control of acromegaly, and screening and management of its comorbidities: expert consensus. Endocrinol Diabetes Nutr (English Ed). 2018;65(5):297‐305. 10.1016/j.endinu.2018.01.008 29653911

[152] Melmed S, Casanueva FF, Klibanski A, et al A consensus on the diagnosis and treatment of acromegaly complications. Pituitary. 2013;16(3):294‐302. 22903574 10.1007/s11102-012-0420-x PMC3730092

[153] Marques P, Sagarribay A, Tortosa F, et al Multidisciplinary team care in pituitary tumours. Cancers (Basel). 2024;16(5):950. 38473312 10.3390/cancers16050950 PMC10930925

[154] Giustina A, Uygur MM, Frara S, et al Standards of care for medical management of acromegaly in pituitary tumor centers of excellence (PTCOE). Pituitary. 2024;27(4):381‐388. 38833044 10.1007/s11102-024-01397-w PMC11289172

[155] Ragonese M, Di Bella G, Spagnolo F, et al Serum NT-pro-BNP levels predict cardiovascular events in acromegaly patients. Exp Clin Endocrinol Diabetes. 2022;130(4):229‐236. 34942671 10.1055/a-1540-5009 PMC9072124

[156] Eichner M, Wallaschofski H, Schminke U, et al Relation of IGF-I with subclinical cardiovascular markers including intima-media thickness, left ventricular mass index and NT-proBNP. Eur J Endocrinol. 2020;182(1):79‐90. 31705797 10.1530/EJE-19-0470

[157] Sherin RPV, Vietor NO, Usman A, Hoang TD, Shakir MKM. Cardiovascular disorders associated with acromegaly: an update. Endocr Pract. 2024;30(12):1212‐1219. 39332498 10.1016/j.eprac.2024.09.014

[158] van der Klaauw AA, Bax JJ, Roelfsema F, et al Uncontrolled acromegaly is associated with progressive mitral valvular regurgitation. Growth Horm IGF Res. 2006;16(2):101‐107. 16580860 10.1016/j.ghir.2006.02.002

[159] Abreu-Lomba A, Aristizábal-Colorado D, Sierra-Castillo S, et al Prevalence and characteristics of heart disease in patients with acromegaly in Colombia (RAPACO HEART study). Cardiol. 2026;151(2):226‐235. Doi: 10.1159/000545875 40349690

[160] Huang R, Jin J, Zhang P, et al Use of speckle tracking echocardiography in evaluating cardiac dysfunction in patients with acromegaly: an update. Front Endocrinol (Lausanne). 2023;14:1260842. 37929035 10.3389/fendo.2023.1260842 PMC10623426

[161] Bozkurt B, Colvin M, Cook J, et al Current diagnostic and treatment strategies for specific dilated cardiomyopathies: a scientific statement from the American Heart Association. Circulation. 2016;134(23):e579‐e646. 27832612 10.1161/CIR.0000000000000455

[162] Warszawski L, Kasuki L, Sá R, et al Low frequency of cardniac arrhythmias and lack of structural heart disease in medically-naïve acromegaly patients: a prospective study at baseline and after 1 year of somatostatin analogs treatment. Pituitary. 2016;19(6):582‐589. 27591859 10.1007/s11102-016-0749-7

[163] Hayward RP, Emanuel RW, Nabarro JDN. Acromegalic heart disease: influence of treatment of the acromegaly on the heart. Q J Med. 1987;62(237):41‐58. 2962220

[164] Çelik IE, Firat SN, Bozkurt U, Yarlioğlu M, Duran M, Murat SN. Assessment of Tp-e interval, Tp-e/QT, and Tp-e/QTc ratios in patients with acromegaly. Turkish J Med Sci. 2022;52(3):809‐815. 10.55730/1300-0144.5377 PMC10390144 36326330

[165] Tekin ZZ, Pamukcu HE, Kayihan S, et al Electrocardiographic ventricular arrhythmia parameters during diagnosis and after the treatment of acromegaly: a case-control study. Heliyon. 2024;10(19):e38033. 39398067 10.1016/j.heliyon.2024.e38033 PMC11471207

[166] Dutta P, Das S, Bhansali A, et al Congestive heart failure in acromegaly: a review of 6 cases. Indian J Endocrinol Metab. 2012;16(6):987‐990. 23226648 10.4103/2230-8210.103007 PMC3510973

[167] Herrmann BL, Bruch C, Saller B, et al Occurrence of ventricular late potentials in patients with active acromegaly. Clin Endocrinol (Oxf). 2001;55(2):201‐207. 11531926 10.1046/j.1365-2265.2001.01319.x

[168] Lombardi G, Colao A, Marzullo P, et al Improvement of left ventricular hypertrophy and arrhythmias after lanreotide-induced GH and IGF-I decrease in acromegaly. A prospective multi-center study. J Endocrinol Invest. 2002;25(11):971‐976. 12553557 10.1007/BF03344070

[169] Melkozerov K V, Przhiyalkovskaya EG, Tarbaeva N V, et al Heart arrhythmias and conduction disorders in patients with acromegaly: the role of cardiac magnetic resonance imaging. Ter Arkh. 2020;92(10):70‐77. 10.26442/00403660.2020.10.000787 33346482

[170] Parolin M, Dassie F, Vettor R, Steeds RP, Maffei P. Electrophysiological features in acromegaly: re-thinking the arrhythmic risk? J Endocrinol Invest. 2021;44(2):209‐221. 32632903 10.1007/s40618-020-01343-0

[171] Paisley AN, Banerjee M, Rezai M, et al Changes in arterial stiffness but not carotid intimal thickness in acromegaly. J Clin Endocrinol Metab. 2011;96(5):1486‐1492. 21346071 10.1210/jc.2010-2225

[172] Sardella C, Cappellani D, Urbani C, et al Disease activity and lifestyle influence comorbidities and cardiovascular events in patients with acromegaly. Eur J Endocrinol. 2016;175(5):443‐453. 27528501 10.1530/EJE-16-0562

[173] Bex M, Abs R, T' et al AcroBel—the Belgian registry on acromegaly: a survey of the “real-life” outcome in 418 acromegalic subjects. Eur J Endocrinol. 2007;157(4):399‐409. 17893253 10.1530/EJE-07-0358

[174] Stocker M, Zimmermann SE, Laager R, et al Cardiovascular risk in patients with acromegaly vs. non-functioning pituitary adenoma following pituitary surgery: an active-comparator cohort study. Pituitary. 2024;27(5):518‐526. 38819619 10.1007/s11102-024-01405-z

[175] Anagnostis P, Efstathiadou ZA, Gougoura S, et al Oxidative stress and reduced antioxidative status, along with endothelial dysfunction in acromegaly. Horm Metab Res. 2013;45(4):314‐318. 23093460 10.1055/s-0032-1323765

[176] Thomas M, Berni E, Jenkins-Jones S, et al Insulin-like growth factor-1, growth hormone and disease outcomes in acromegaly: a population study. Clin Endocrinol (Oxf). 2021;95(1):143‐152. 33749903 10.1111/cen.14468

[177] Cannavo S, Almoto B, Cavalli G, et al Acromegaly and coronary disease: an integrated evaluation of conventional coronary risk factors and coronary calcifications detected by computed tomography. J Clin Endocrinol Metab. 2006;91(10):3766‐3772. 16835284 10.1210/jc.2005-2857

[178] dos Santos Silva CM, Lima GAB, Volschan ICM, et al Low risk of coronary artery disease in patients with acromegaly. Endocrine. 2015;50(3):749‐755. 25982151 10.1007/s12020-015-0628-4

[179] Vrints C, Andreotti F, Koskinas KC, et al 2024 ESC Guidelines for the management of chronic coronary syndromes. Eur Heart J. 2024;45(36):3415‐3537. 39210710 10.1093/eurheartj/ehae177

[180] Ayuk J, Clayton RN, Holder G, Sheppard MC, Stewart PM, Bates AS. Growth hormone and pituitary radiotherapy, but not serum insulin-like growth factor-I concentrations, predict excess mortality in patients with acromegaly. J Clin Endocrinol Metab. 2004;89(4):1613‐1617. 15070920 10.1210/jc.2003-031584

[181] Ntali G, Karavitaki N. Efficacy and complications of pituitary irradiation. Endocrinol Metab Clin North Am. 2015;44(1):117‐126. 25732648 10.1016/j.ecl.2014.10.009

[182] van Westrhenen A, Muskens IS, Verhoeff JJC, Smith TRS, Broekman MLD. Ischemic stroke after radiation therapy for pituitary adenomas: a systematic review. J Neurooncol. 2017;135(1):1‐11. 28660317 10.1007/s11060-017-2530-9 PMC5658475

[183] Petrossians P, Daly AF, Natchev E, et al Acromegaly at diagnosis in 3173 patients from the Liège Acromegaly Survey (LAS) database. Endocr Relat Cancer. 2017;24(10):505‐518. 28733467 10.1530/ERC-17-0253 PMC5574208

[184] Reid TJ, Post KD, Bruce JN, Nabi Kanibir M, Reyes-Vidal CM, Freda PU. Features at diagnosis of 324 patients with acromegaly did not change from 1981 to 2006: acromegaly remains under-recognized and under-diagnosed. Clin Endocrinol (Oxf). 2010;72(2):203‐208. 19473180 10.1111/j.1365-2265.2009.03626.x PMC2866138

[185] Osorio RC, Oh JY, Choudhary N, Lad M, Savastano L, Aghi MK. Pituitary adenomas and cerebrovascular disease: a review on pathophysiology, prevalence, and treatment. Front Endocrinol (Lausanne). 2022;13:1064216. 36578965 10.3389/fendo.2022.1064216 PMC9791098

[186] Erfurth EM, Hagmar L. Cerebrovascular disease in patients with pituitary tumors. Trends Endocrinol Metab. 2005;16(7):334‐342. 16054833 10.1016/j.tem.2005.07.004

[187] Oshino S, Nishino A, Suzuki T, et al Prevalence of cerebral aneurysm in patients with acromegaly. Pituitary. 2013;16(2):195‐201. 22752346 10.1007/s11102-012-0404-x

[188] Vlak MHM, Algra A, Brandenburg R, Rinkel GJE. Prevalence of unruptured intracranial aneurysms, with emphasis on sex, age, comorbidity, country, and time period: a systematic review and meta-analysis. Lancet Neurol. 2011;10(7):626‐636. 21641282 10.1016/S1474-4422(11)70109-0

[189] Alnaaim SA, Al-kuraishy HM, Zailaie MM, et al The potential link between acromegaly and risk of acute ischemic stroke in patients with pituitary adenoma: a new perspective. Acta Neurol Belg. 2024;124(3):755‐766. 37584889 10.1007/s13760-023-02354-3 PMC11139727

[190] Fleseriu M, Biller BMK, Freda PU, et al A Pituitary Society update to acromegaly management guidelines. Pituitary. 2021;24(1):1‐13. 33079318 10.1007/s11102-020-01091-7 PMC7864830

[191] D’Agostino RB, Vasan RS, Pencina MJ, et al General cardiovascular risk profile for use in primary care: the Framingham heart study. Circulation. 2008;117(6):743‐753. 18212285 10.1161/CIRCULATIONAHA.107.699579

[192] Elarabi AM, Mosleh E, Alamlih LI, Albakri MM, Ibrahim WH. Massive pulmonary embolism as the initial presentation of acromegaly: is acromegaly a hypercoagulable condition? Am J Case Rep. 2018;19:1541‐1545. 30593586 10.12659/AJCR.911572 PMC6322059

[193] Al-Anbagi U, Usman S, Saad A, Nashwan AJ. Unexplained massive pulmonary embolism in acromegaly patient: a case report. Clin Case Reports. 2024;12(5):e8867. 10.1002/ccr3.8867 PMC11087215 38736578

[194] Mendoza E, Lou MC, Tanchee-Ngo MJ, Mercado-Asis L. Acromegaly with cardiomyopathy, cardiac thrombus and hemorrhagic cerebral infarct: a case report of therapeutic dilemma with review of literature. Int J Endocrinol Metab. 2015;13(2):e18841. 25926851 10.5812/ijem.18841 PMC4397949

[195] Mestrón A, Webb SM, Astorga R, et al Epidemiology, clinical characteristics, outcome, morbidity and mortality in acromegaly based on the Spanish Acromegaly Registry (Registro Español de Acromegalia, REA). Eur J Endocrinol. 2004;151(4):439‐446. 15476442 10.1530/eje.0.1510439

[196] Criado-García J . Prophylaxis of venous thromboembolism in medical patients. Rev Clin Esp. 2020;220(1):1‐9. 32958201 10.1016/j.rce.2020.07.005

[197] Dreval AV, Trigolosova IV, Misnikova IV, et al Prevalence of diabetes mellitus in patients with acromegaly. Endocr Connect. 2014;3(2):93‐98. 24692509 10.1530/EC-14-0021 PMC5327845

[198] Rolla M, Jawiarczyk-Przybyłowska A, Halupczok-Żyła J, et al Complications and comorbidities of acromegaly—retrospective study in Polish center. Front Endocrinol (Lausanne). 2021;12:642131. 33796075 10.3389/fendo.2021.642131 PMC8009182

[199] Gatto F, Trifirò G, Lapi F, et al Epidemiology of acromegaly in Italy: analysis from a large longitudinal primary care database. Endocrine. 2018;61(3):533‐541. 29797214 10.1007/s12020-018-1630-4

[200] Malchiodi E, Arosio M, Borraccino A, et al Epidemiology of acromegaly in Italy. Endocr Abstr. 2010;22:P620.

[201] Fieffe S, Morange I, Petrossians P, et al Diabetes in acromegaly, prevalence, risk factors, and evolution: data from the French Acromegaly Registry. Eur J Endocrinol. 2011;164(6):877‐884. 21464140 10.1530/EJE-10-1050

[202] Cordido F, García Arnés JA, Marazuela Aspiroz M, Torres Vela E. Guía práctica de diagnóstico y tratamiento de la acromegalia. Endocrinol Nutr. 2013;60(8):457.e1‐457.e15. 10.1016/j.endonu.2013.01.012 23660006

[203] Committee ADAPP, ElSayed NA, McCoy RG, et al 2. Diagnosis and classification of diabetes: standards of care in diabetes—2025. Diabetes Care. 2025;48(Suppl 1):S27‐S49. 39651986 10.2337/dc25-S002 PMC11635041

[204] Störmann S, Meyhöfer SM, Groener JB, et al Management of pasireotide-induced hyperglycemia in patients with acromegaly: an experts’ consensus statement. Front Endocrinol (Lausanne). 2024;15:1348990. 38405148 10.3389/fendo.2024.1348990 PMC10884330

[205] Costanza F, Basile C, Chiloiro S, et al Impact of pasireotide on lipid and glucose metabolism in patients with acromegaly: a systematic review and meta-analysis. J Endocrinol Invest. 2025;48(12):2799‐2812. 40622518 10.1007/s40618-025-02642-0 PMC12640336

[206] Romanisio M, Pitino R, Ferrero A, et al Discordant biochemical parameters of acromegaly remission do not influence the prevalence or aggressiveness of metabolic comorbidities: a single-center study. Front Endocrinol (Lausanne). 2023;14:1256975. 37829686 10.3389/fendo.2023.1256975 PMC10565344

[207] Tan KCB, Shiu SWM, Janus ED, Lam KSL. LDL subfractions in acromegaly: relation to growth hormone and insulin-like growth factor-I. Atherosclerosis. 1997;129(1):59‐65. 9069518 10.1016/s0021-9150(96)06015-7

[208] Schutte AE, Volpe M, Tocci G, Conti E. Revisiting the relationship between blood pressure and insulin-like growth factor-1. Hypertension. 2014;63(5):1070‐1077. 24566078 10.1161/HYPERTENSIONAHA.113.03057

[209] Mach F, Koskinas KC, van Lennep JE R, et al 2025 Focused update of the 2019 ESC/EAS Guidelines for the management of dyslipidaemias. Atherosclerosis. 2025;409:120479. 40885687 10.1016/j.atherosclerosis.2025.120479

[210] Pivonello R, Auriemma RS, Delli Veneri A, et al Global psychological assessment with the evaluation of life and sleep quality and sexual and cognitive function in a large number of patients with acromegaly: a cross-sectional study. Eur J Endocrinol. 2022;187(6):823‐845. 36165745 10.1530/EJE-22-0263 PMC9782455

[211] Zhao X, Heng L, Qu Y, et al Densely granulated adenoma pattern is associated with an increased risk of obstructive sleep apnea in patients with acromegaly. Sleep Breath. 2022;26(3):1381‐1387. 34383277 10.1007/s11325-021-02468-z

[212] Parolin M, Dassie F, Alessio L, et al Obstructive sleep apnea in acromegaly and the effect of treatment: a systematic review and meta-analysis. J Clin Endocrinol Metab. 2020;105(3):dgz116. 31722411 10.1210/clinem/dgz116

[213] Galerneau LM, Pépin JL, Borel AL, et al Acromegaly in sleep apnoea patients: a large observational study of 755 patients. Eur Respir J. 2016;48(5):1489‐1492. 27660510 10.1183/13993003.01229-2016

[214] Sesmilo G, Resmini E, Sambo M, et al Prevalence of acromegaly in patients with symptoms of sleep apnea. PLoS One. 2017;12(9):e0183539. 28898247 10.1371/journal.pone.0183539 PMC5595301

[215] Heinrich DA, Reinholz C, Bauer M, et al IGF-1-based screening reveals a low prevalence of acromegaly in patients with obstructive sleep apnea. Endocrine. 2018;60(2):317‐322. 29388045 10.1007/s12020-018-1538-z

[216] Epstein LJ, Kristo D, Strollo PJ, et al Clinical guideline for the evaluation, management and long-term care of obstructive sleep apnea in adults. J Clin Sleep Med. 2009;5(3):263‐276. 19960649 PMC2699173

[217] Giustina A, Mancini T, Boscani PF, et al Assessment of the awareness and management of cardiovascular complications of acromegaly in Italy. The COM.E.T.A. (COMorbidities evaluation and treatment in acromegaly) study. J Endocrinol Invest. 2008;31(8):731‐738. 18852535 10.1007/BF03346423

[218] Sze L, Schmid C, Bloch KE, Bernays R, Brändle M. Effect of transsphenoidal surgery on sleep apnoea in acromegaly. Eur J Endocrinol. 2007;156(3):321‐329. 17322492 10.1530/eje.1.02340

[219] Giustina A, Biermasz N, Casanueva FF, et al Consensus on criteria for acromegaly diagnosis and remission. Pituitary. 2023;27(1):7‐22. 37923946 10.1007/s11102-023-01360-1 PMC10837217

[220] Cardoso LM, Marques P, Pereira MT, et al Diagnosis and management of acromegaly: a consensus statement of the pituitary study group of the Portuguese Society of Endocrinology, Diabetes and Metabolism. Endocrinol Insights. 2025;20:29‐58.

[221] Giustina A, Barkhoudarian G, Beckers A, et al Multidisciplinary management of acromegaly: a consensus. Rev Endocr Metab Disord. 2020;21(4):667‐678. 32914330 10.1007/s11154-020-09588-z PMC7942783

[222] Dimitriadis GK, Angelousi A, Weickert MO, Randeva HS, Kaltsas G, Grossman A. Paraneoplastic endocrine syndromes. Endocr Relat Cancer. 2017;24(6):R173‐R190. 28341725 10.1530/ERC-17-0036

[223] Puglisi S, Terzolo M. Hypertension and acromegaly. Endocrinol Metab Clin North Am. 2019;48(4):779‐793. 31655776 10.1016/j.ecl.2019.08.008

[224] Pirchio R, Auriemma RS, Vergura A, Pivonello R, Colao A. Long-term pasireotide therapy in acromegaly: extensive real-life experience of a referral center. J Endocrinol Invest. 2024;47(8):1887‐1901. 38532073 10.1007/s40618-023-02299-7 PMC11266387

[225] Urbani C, Dassie F, Zampetti B, et al Real-life data of pasireotide LAR in acromegaly: a long-term follow-up. J Endocrinol Invest. 2024;47(7):1733‐1741. 38244140 10.1007/s40618-023-02275-1 PMC11196287

[226] Popa Ilie IR, Dobrea CM, Butuca A, et al Real-life data on the safety of pasireotide in acromegaly: insights from EudraVigilance. Pharmaceuticals (Basel). 2024;17(12):1631. 39770473 10.3390/ph17121631 PMC11728653

[227] Araujo-Castro M, Biagetti B, Torre EM, et al Pegvisomant and pasireotide in PRL and GH co-secreting vs GH-secreting Pit-NETs. Endocr Relat Cancer. 2024;31(7):e240043. 38713182 10.1530/ERC-24-0043

[228] Breitschaft A, Hu K, Darstein C, et al Effects of subcutaneous pasireotide on cardiac repolarization in healthy volunteers: a single-center, phase i, randomized, four-way crossover study. J Clin Pharmacol. 2014;54(1):75‐86. 24242903 10.1002/jcph.213 PMC4272414

[229] Berg C, Petersenn S, Lahner H, et al Cardiovascular risk factors in patients with uncontrolled and long-term acromegaly: comparison with matched data from the general population and the effect of disease control. J Clin Endocrinol Metab. 2010;95(8):3648‐3656. 20463098 10.1210/jc.2009-2570

[230] Colao A, Terzolo M, Bondanelli M, et al GH and IGF-I excess control contributes to blood pressure control: results of an observational, retrospective, multicentre study in 105 hypertensive acromegalic patients on hypertensive treatment. Clin Endocrinol (Oxf). 2008;69(4):613‐620. 18410555 10.1111/j.1365-2265.2008.03258.x

[231] Yonenaga M, Fujio S, Habu M, et al Postoperative changes in metabolic parameters of patients with surgically controlled acromegaly: assessment of new stringent cure criteria. Neurol Med Chir (Tokyo). 2018;58(4):147‐155. 29479042 10.2176/nmc.oa.2017-0215 PMC5929912

[232] Reyes-Vidal C, Fernandez JC, Bruce JN, et al Prospective study of surgical treatment of acromegaly: effects on ghrelin, weight, adiposity, and markers of CV risk. J Clin Endocrinol Metab. 2014;99(11):4124‐4132. 25137427 10.1210/jc.2014-2259 PMC4223431

[233] Serri O, Beauregard C, Hardy J. Long-term biochemical Status and disease-related morbidity in 53 postoperative patients with acromegaly. J Clin Endocrinol Metab. 2004;89(2):658‐661. 14764777 10.1210/jc.2003-030915

[234] Colao A, Ferone D, Marzullo P, et al Long-term effects of depot long-acting somatostatin analog octreotide on hormone levels and tumor mass in acromegaly. J Clin Endocrinol Metab. 2001;86(6):2779‐2786. 11397887 10.1210/jcem.86.6.7556

[235] Colao A, Auriemma RS, Galdiero M, Lombardi G, Pivonello R. Effects of initial therapy for five years with somatostatin analogs for acromegaly on growth hormone and insulin-like growth factor-I levels, tumor shrinkage, and cardiovascular disease: a prospective study. J Clin Endocrinol Metab. 2009;94(10):3746‐3756. 19622615 10.1210/jc.2009-0941

[236] De Martino MC, Auriemma RS, Brevetti G, et al The treatment with growth hormone receptor antagonist in acromegaly: effect on vascular structure and function in patients resistant to somatostatin analogues. J Endocrinol Invest. 2010;33(9):663‐670. 20595800 10.1007/BF03346667

[237] Auriemma RS, Grasso LFS, Galdiero M, et al Effects of long-term combined treatment with somatostatin analogues and pegvisomant on cardiac structure and performance in acromegaly. Endocrine. 2017;55(3):872‐884. 27295183 10.1007/s12020-016-0995-5

[238] Pivonello R, Galderisi M, Auriemma RS, et al Treatment with growth hormone receptor antagonist. J Clin Endocrinol Metab. 2007;92(2):476‐482. 17105844 10.1210/jc.2006-1587

[239] Ford ES . Trends in mortality from all causes and cardiovascular disease among hypertensive and nonhypertensive adults in the United States. Circulation. 2011;123(16):1737‐1744. 21518989 10.1161/CIRCULATIONAHA.110.005645

[240] Pirchio R, Auriemma RS, Montini ME, Vergura A, Pivonello R, Colao A. Control of acromegaly in more than 90% of patients after 10 years of pegvisomant therapy: an European referral centre real-life experience. J Endocrinol Invest. 2023;46(5):1027‐1038. 36892739 10.1007/s40618-022-01980-7 PMC10105681

[241] Tachibana H, Yamaguchi H, Abe S, et al Improvement of ventricular arrhythmia by octreotide treatment in acromegalic cardiomyopathy. Jpn Heart J. 2003;44(6):1027‐1031. 14711197 10.1536/jhj.44.1027

[242] Suyama K, Uchida D, Tanaka T, et al Octreotide improved ventricular arrhythmia in an acromegalic patient. Endocr J. 2000;47(Suppl):S73‐S75. 10890189 10.1507/endocrj.47.supplmarch_s73

[243] Auriemma RS, Pivonello R, De Martino MC, et al Treatment with GH receptor antagonist in acromegaly: effect on cardiac arrhythmias. Eur J Endocrinol. 2013;168(1):15‐22. 23065994 10.1530/EJE-12-0596

[244] Maione L, Garcia C, Bouchachi A, et al No evidence of a detrimental effect of cabergoline therapy on cardiac valves in patients with acromegaly. J Clin Endocrinol Metab. 2012;97(9):E1714‐E1719. 22723314 10.1210/jc.2012-1833

[245] Maffei P, Martini C, Milanesi A, et al Late potentials and ventricular arrhythmias in acromegaly. Int J Cardiol. 2005;104(2):197‐203. 16168814 10.1016/j.ijcard.2004.12.010

[246] Kuhn E, Maione L, Bouchachi A, et al Long-term effects of pegvisomant on comorbidities in patients with acromegaly: a retrospective single-center study. Eur J Endocrinol. 2015;173(5):693‐702. 26429918 10.1530/EJE-15-0500 PMC4592912

[247] Popielarz-Grygalewicz A, Gasior JS, Konwicka A, et al Heart in acromegaly: the echocardiographic characteristics of patients diagnosed with acromegaly in various stages of the disease. Int J Endocrinol. 2018;2018:6935054. 30123265 10.1155/2018/6935054 PMC6079421

[248] Cozzolino A, Feola T, Simonelli I, et al Somatostatin analogs and glucose metabolism in acromegaly: a meta-analysis of prospective interventional studies. J Clin Endocrinol Metab. 2018;103(6):2089‐2099. 10.1210/jc.2017-02566 29590371

[249] Colao A, Auriemma RS, Savastano S, et al Glucose tolerance and somatostatin analog treatment in acromegaly: a 12-month study. J Clin Endocrinol Metab. 2009;94(8):2907‐2914. 19491229 10.1210/jc.2008-2627

[250] Biagetti B, Araujo-Castro M, Tebe C, Marazuela M, Puig-Domingo M. Real-world evidence of effectiveness and safety of pasireotide in the treatment of acromegaly: a systematic review and meta-analysis. Rev Endocr Metab Disord. 2025;26(1):97‐111. 39527181 10.1007/s11154-024-09928-3 PMC11790789

[251] Gadelha M, Bex M, Colao A, et al Evaluation of the efficacy and safety of switching to pasireotide in patients with acromegaly inadequately controlled with first-generation somatostatin analogs. Front Endocrinol (Lausanne). 2020;10:931. 32117045 10.3389/fendo.2019.00931 PMC7008501

[252] Tahara S, Murakami M, Kaneko T, et al Efficacy and safety of long-acting pasireotide in Japanese patients with acromegaly or pituitary gigantism: results from a multicenter, open-label, randomized, phase 2 study. Endocr J. 2017;64(7):735‐747. 28592706 10.1507/endocrj.EJ16-0624

[253] Wolf P, Dormoy A, Maione L, et al Impairment in insulin secretion without changes in insulin resistance explains hyperglycemia in patients with acromegaly treated with pasireotide LAR. Endocr Connect. 2022;11(12):e220296. 36269605 10.1530/EC-22-0296 PMC9716376

[254] Gadelha MR, Gu F, Bronstein MD, et al Risk factors and management of pasireotide-associated hyperglycemia in acromegaly. Endocr Connect. 2020;9(12):1178‐1190. 33434154 10.1530/EC-20-0361 PMC7774766

[255] Samson SL, Gu F, Feldt-Rasmussen U, et al Managing pasireotide-associated hyperglycemia: a randomized, open-label, phase IV study. Pituitary. 2021;24(6):887‐903. 34275099 10.1007/s11102-021-01161-4 PMC8550309

[256] Feola T, Cozzolino A, Simonelli I, et al Pegvisomant improves glucose metabolism in acromegaly: a meta-analysis of prospective interventional studies. J Clin Endocrinol Metab. 2019;104(7):2892‐2902. 30869797 10.1210/jc.2018-02281

[257] Lindberg-Larsen R, Møller N, Schmitz O, et al The impact of pegvisomant treatment on substrate metabolism and insulin sensitivity in patients with acromegaly. J Clin Endocrinol Metab. 2007;92(5):1724‐1728. 17341562 10.1210/jc.2006-2276

[258] Higham CE, Rowles S, Russell-Jones D, Umpleby AM, Trainer PJ. Pegvisomant improves insulin sensitivity and reduces overnight free fatty acid concentrations in patients with acromegaly. J Clin Endocrinol Metab. 2009;94(7):2459‐2463. 19366854 10.1210/jc.2008-2086

[259] Lopez Vicchi F, Luque GM, Brie B, Nogueira JP, Garcia Tornadu I, Becu-Villalobos D. Dopaminergic drugs in type 2 diabetes and glucose homeostasis. Pharmacol Res. 2016;109:74‐80. 26748034 10.1016/j.phrs.2015.12.029

[260] Rau H, Althoff PH, Schmidt K, Badenhoop K, Usadel KH. Bromocriptine treatment over 12 years in acromegaly: effect on glucose tolerance and insulin secretion. Clin Investig. 1993;71(5):372‐378. 10.1007/BF00186626 8508007

[261] Roemmler J, Steffin B, Gutt B, et al The acute effect of a single application of cabergoline on endogenous GH levels in patients with acromegaly on pegvisomant treatment. Growth Horm IGF Res. 2010;20(5):338‐344. 20598600 10.1016/j.ghir.2010.05.004

[262] Varaldo E, Prencipe N, Bona C, et al Effect of cabergoline on weight and glucose metabolism in patients with acromegaly. J Endocrinol Invest. 2024;47(12):3019‐3028. 38787507 10.1007/s40618-024-02396-1 PMC11549174

[263] Mishra M, Durrington P, Mackness M, et al The effect of atorvastatin on serum lipoproteins in acromegaly. Clin Endocrinol (Oxf). 2005;62(6):650‐655. 15943824 10.1111/j.1365-2265.2005.02273.x

[264] Shao XQ, Chen ZY, Wang M, et al Effects of long-acting somatostatin analogues on lipid metabolism in patients with newly diagnosed acromegaly: a retrospective study of 120 cases. Horm Metab Res. 2022;54(1):25‐32. 34986497 10.1055/a-1717-9332

[265] Sesmilo G, Fairfield WP, Katznelson L, et al Cardiovascular risk factors in acromegaly before and after normalization of serum IGF-I levels with the GH antagonist pegvisomant. J Clin Endocrinol Metab. 2002;87(4):1692‐1699. 11932303 10.1210/jcem.87.4.8364

[266] Dzialach L, Respondek W, Witek P. Real-world experience with pasireotide-LAR in Cushing's disease: single-center 12-month observational study. J Clin Med. 2025;14(8):2794. 40283624 10.3390/jcm14082794 PMC12028122

[267] Cho J, Kim JH, Kim YH, Lee J. Obstructive sleep apnea screening and effects of surgery in acromegaly: a prospective study. Endocrinol Metab (Seoul). 2024;39(4):641‐652. 38918903 10.3803/EnM.2024.1933 PMC11375303

[268] Herrmann BL, Wessendorf TE, Ajaj W, Kahlke S, Teschler H, Mann K. Effects of octreotide on sleep apnoea and tongue volume (magnetic resonance imaging) in patients with acromegaly. Eur J Endocrinol. 2004;151(3):309‐315. 15362959 10.1530/eje.0.1510309

[269] Ip MSM, Tan KCB, Peh WCG, Lam KSL. Effect of sandostatin LAR on sleep apnoea in acromegaly: correlation with computerized tomographic cephalometry and hormonal activity. Clin Endocrinol (Oxf). 2001;55(4):477‐483. 11678830 10.1046/j.1365-2265.2001.01358.x

[270] Annamalai AK, Webb A, Kandasamy N, et al A comprehensive study of clinical, biochemical, radiological, vascular, cardiac, and sleep parameters in an unselected cohort of patients with acromegaly undergoing presurgical somatostatin receptor ligand therapy. J Clin Endocrinol Metab. 2013;98(3):1040‐1050. 23393175 10.1210/jc.2012-3072

[271] Berg C, Wessendorf TE, Mortsch F, et al Influence of disease control with pegvisomant on sleep apnoea and tongue volume in patients with active acromegaly. Eur J Endocrinol. 2009;161(6):829‐835. 19773369 10.1530/EJE-09-0694

[272] American Diabetes Association Professional Practice Committee . 5. Facilitating positive health behaviors and well-being to improve health outcomes: standards of care in diabetes—2025. Diabetes Care. 2025;48(1 Suppl 1):S86‐S127. 39651983 10.2337/dc25-S005 PMC11635047

[273] Padwal R, Laupacis A. Antihypertensive therapy and incidence of type 2 diabetes: a systematic review. Diabetes Care. 2004;27(1):247‐255. 14693997 10.2337/diacare.27.1.247

[274] Fleseriu M, Langlois F, Lim DST, Varlamov EV, Melmed S. Acromegaly: pathogenesis, diagnosis, and management. Lancet Diabetes Endocrinol. 2022;10(11):804‐826. 36209758 10.1016/S2213-8587(22)00244-3

[275] Zaina A, Prencipe N, Golden E, et al How to position sodium-glucose co-transporter 2 inhibitors in the management of diabetes in acromegaly patients. Endocrine. 2023;80(3):491‐499. 37000406 10.1007/s12020-023-03352-4

[276] Quarella M, Walser D, Brändle M, Fournier JY, Bilz S. Rapid onset of diabetic ketoacidosis after SGLT2 inhibition in a patient with unrecognized acromegaly. J Clin Endocrinol Metab. 2017;102(5):1451‐1453. 28323955 10.1210/jc.2017-00082

[277] Gatto F, Arecco A, Amarù J, et al Differential impact of medical therapies for acromegaly on glucose metabolism. Int J Mol Sci. 2025;26(2):465. 39859181 10.3390/ijms26020465 PMC11764544

[278] American Diabetes Association Professional Practice Committee . 9. Pharmacologic approaches to glycemic treatment: standards of care in diabetes-2025. Diabetes Care. 2025;48(1 Suppl 1):S181‐S206. 39651989 10.2337/dc25-S009 PMC11635045

